# A Model of Fast Hebbian Spike Latency Normalization

**DOI:** 10.3389/fncom.2017.00033

**Published:** 2017-05-15

**Authors:** Hafsteinn Einarsson, Marcelo M. Gauy, Johannes Lengler, Angelika Steger

**Affiliations:** ^1^Department of Computer Science, Institute of Theoretical Computer Science, ETH ZurichZurich, Switzerland; ^2^Collegium HelveticumZurich, Switzerland

**Keywords:** homeostasis, STDP, oscillations, synchrony, synapse memory, metaplasticity

## Abstract

Hebbian changes of excitatory synapses are driven by and enhance correlations between pre- and postsynaptic neuronal activations, forming a positive feedback loop that can lead to instability in simulated neural networks. Because Hebbian learning may occur on time scales of seconds to minutes, it is conjectured that some form of fast stabilization of neural firing is necessary to avoid runaway of excitation, but both the theoretical underpinning and the biological implementation for such homeostatic mechanism are to be fully investigated. Supported by analytical and computational arguments, we show that a Hebbian spike-timing-dependent metaplasticity rule, accounts for inherently-stable, quick tuning of the total input weight of a single neuron in the general scenario of asynchronous neural firing characterized by UP and DOWN states of activity.

## 1. Introduction

Since the discovery of long-term synaptic plasticity by Bliss and Lømo (1973), Hebb's postulate that “cells that fire together wire together” (Hebb, 1949; Schatz, 1992) has become the prominent hypothesis whereby the brain learns and forms new memories. Generally speaking, Hebbian learning may refer to any change of efficacy of synaptic transmission (i.e., the “synaptic weight”) by synaptic plasticity that solely depends on correlations between firing activities of pre- and postsynaptic neurons (Abbott and Nelson, 2000). In this regard, spike-timing–dependent plasticity (STDP) may be considered the experimental hallmark of Hebbian learning, insofar as it allows a synapse to be potentiated by correlated pre/post spike pairs while being depressed by correlated post/pre spike pair (Markram et al., 1997; Bi and Poo, 1998).

Despite being appealing for its simple formulation, Hebbian plasticity, and thus STDP, are prone to instability. Depressed synapses tend to become further depressed, and vice-versa, potentiated synapses tend to grow even stronger (Sjöström et al., 2008). *Ad-hoc* mechanisms that compensate for such instabilities are hypothesized to coexist with Hebbian plasticity. Collectively, these mechanisms are known as homeostatic plasticity and are experimentally known to prevent runaway of excitation of single neurons, thereby maintaining a stable level of firing activity (Turrigiano, 2008).

Homeostatic plasticity results in compensatory changes in the overall synaptic drive (e.g., synaptic scaling Turrigiano et al., 1998), changes in the neuronal excitability (intrinsic plasticity Desai, 2003) or changes to the plasticity rules themselves by metaplasticity (Abraham and Bear, 1996; Abraham, 2008). All these experimentally-found homeostatic mechanisms have a relatively slow response compared to rapid plasticity, which is thought necessary for learning. While synaptic weights can change on the timescale of seconds to minutes (Markram et al., 1997; Bi and Poo, 1998; Sjöström et al., 2008), noticeable changes caused by homeostasis generally take hours or even days (Turrigiano et al., 1998; Turrigiano, 1999; Turrigiano and Nelson, 2004; Watt and Desai, 2010). As a consequence, it has been conjectured that a further, fast form of homeostatic plasticity, acting on time scales comparable to those of learning, must exist to maintain firing stability (Zenke et al., 2013; Yger and Gilson, 2015) although its biophysical correlates remain to be explored.

Here we address this conundrum by exploring the requirements for stability of Hebbian learning in the context of rhythmic activity which alternates between high and low rate periods. We refer to these periods as UP and DOWN phases. In this regard, we show by analytical arguments and numerical simulations that stability of activity and weight normalization can be an emergent property of Hebbian plasticity through postsynaptic spike latency normalization (SLN) with respect to the onset of an UP phase of activity. We introduce a Hebbian STDP-based metaplasticity rule, which we refer to as the SLN rule, that includes online estimation of the total synaptic input per neuron by making use of the transitions between UP and DOWN phases. These phases can either be compared to the scenario, ubiquitous in the brain (Gray and McCormick, 1996; Lesica and Stanley, 2004; Engel et al., 2016), of activity that varies strongly and abruptly over time or to long UP and DOWN phases that occur during sleep (Steriade et al., 2001). The effect of sleep on plasticity and homeostasis is not completely established. However, the synaptic homeostasis hypothesis (SHY) considers that synaptic potentation resulting from increased neuronal and synaptic activity by sensory stimulation during wakefulness (Vyazovskiy et al., 2008; Liu et al., 2010; Bushey et al., 2011; Maret et al., 2011) must be downscaled during sleep to re-equilibrate the brain's energy demand, but the homeostatic mechanism for such rescaling is not understood (Tononi and Cirelli, 2003, 2006, 2014). Remarkably, the SLN rule results in fast weight normalization for short UP and DOWN phases which makes it a candidate for a fast homeostasis mechanism during periods of wakefulness whereas, for long UP phases, it results in rescaling of the weights at a lower level in agreement with SHY. We discuss the biophysical correlates and advantages of this rule with respect to other models.

## 2. Materials and methods

### 2.1. Neuronal models

We study two models which differ at their level of abstraction and we characterize them by the type of neurons which are specific to each model. We consider integrate and fire neurons either without (SIF) or with leak (LIF). Hence, to distinguish the models, we refer to them as “the model without leak” or “the model with leak.” When describing the models we adopt the convention to first describe aspects which are common to both models along with minor differences. We present then details pertaining to the model without leak followed by those of the model with leak. In both models, a neuron fires an action potential when its membrane potential υ(*t*) reaches a threshold θ_υ_, after which it is reset to *V*_*r*_, and held to this reset potential for a refractory period τ_ref_. The subthreshold dynamics of υ(*t*) thus evolves according to:

(1) $$\begin{array}{l} C_{m}\frac{d}{dt}\upsilon =g\left(\upsilon \right)+I_{\text{syn}}, \end{array}$$

where *g*(υ) is a voltage dependent term and *I*_syn_ is a term which captures synaptic input. Most results concern binary synapses and therefore we differentiate between weak (*w*) and strong (*s*) synapses whose weights we respectively denote by *w*_*w*_ and *w*_*s*_.

#### 2.1.1. Model without leak

**Figure 1:**
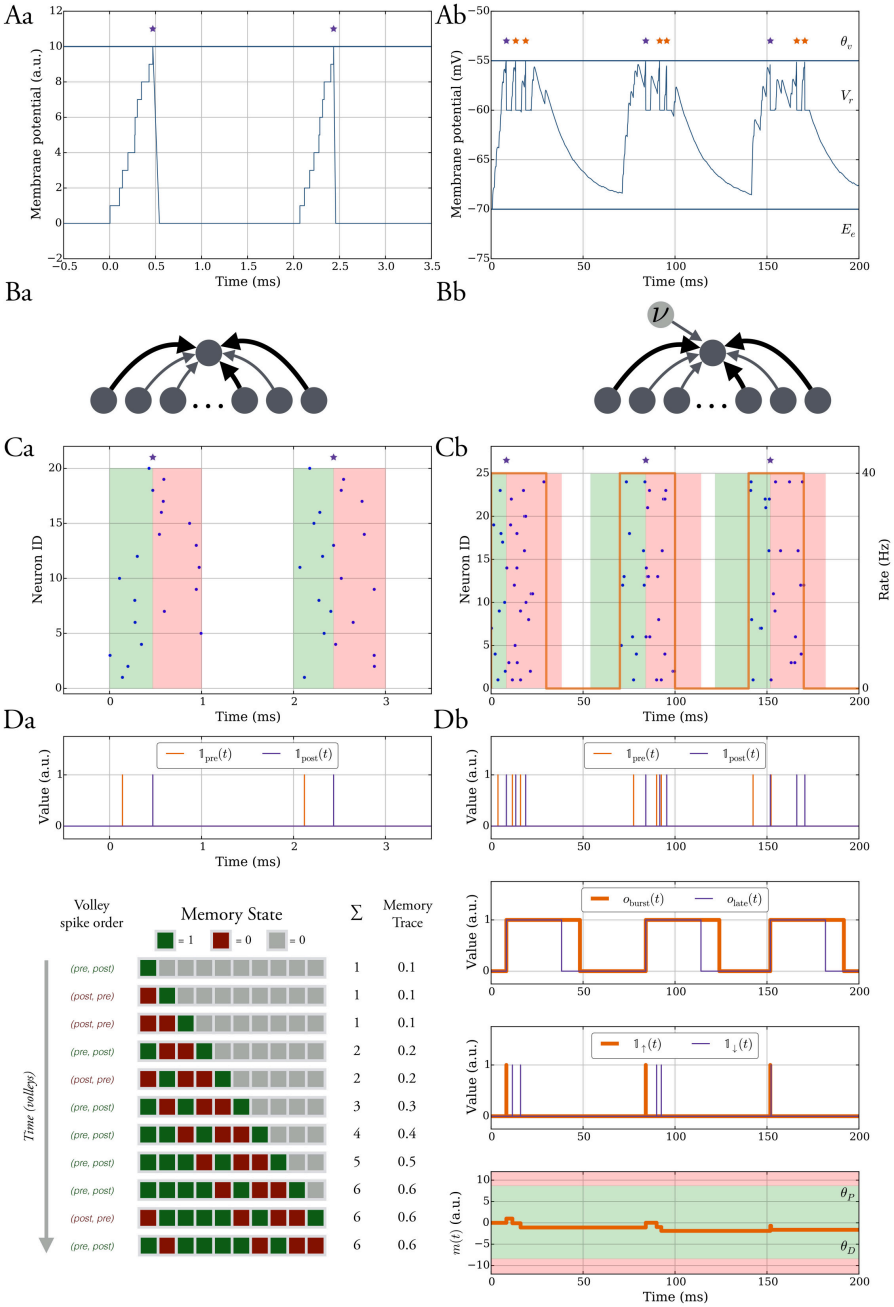
**The model without leak (left column) vs. the model with leak (right column). (A)** In both models the neurons spike when their membrane potential reaches a threshold θ_*v*_ after which it is reset to a value *V*_*r*_ and held at that value for τ_ref_ ms. In the model without leak **(a)**, the neuron receives current-based input whereas in the model with leak **(b)**, the input is conductance-based and the membrane potential decays exponentially. The spikes are shown as stars. We refer to the *purple* spikes as “distinguished” spikes (DSPs). They block other postsynaptic spikes from receiving that label for a period of *T*_burst_ ms. **(B)** Network models. A single target neuron that receives input from a population **(a)**. In the model with leak **(b)**, the neuron additionally receives constant noise input with weight *w*_*n*_ at rate ν. The synapses are binary and can be either weak (*light gray*), with weight *w*_*w*_, or strong, with weight *w*_*s*_ (*dark gray*). **(C)** Input spike model. **(a)** Each input neuron spikes once at a time chosen uniformly at random within a short interval. Presynaptic spikes which occur before (after) the postsynaptic spike lead to a potentiation (depression) signal which is highlighted with a *green* (*red*) shaded region. **(b)** The input spikes are modeled as an inhomogeneous Poisson process. The input neurons switch between a high rate UP phase and a low rate DOWN phase (*orange curve* with respect to right y-axis). The presynaptic spikes which occur in a window before (after) the distinguished postsynaptic spike trigger potentiation (depression) signals and the window is highlighted in *green* (*red*). **(D)** Learning rule. Each synapse has a memory trace *m*(*t*) that is modified for certain spike pair events, similar to standard STDP. **(a)** Potentiation and depression signals are always with respect to spike pairs and we track the spikes with the variables 𝟙_pre_ and 𝟙_post_. The memory in the model without leak contains the last *M* potentiation and depression signals and the memory trace is the fraction of potentiation signals. **(b)** Variables to define distinguished spikes (DSPs), potentiation and depression signals, and the memory trace *m*(*t*) (see Section 2.3 for details).

In Equation (1) we set *g*(υ) = 0 and the input current is the sum of current-based synaptic inputs. A spike of neuron *j* in the input population *I* at time *t*_*j*_ arrives at the postsynaptic neuron without delay and is modeled by a Dirac delta scaled by the synaptic weight *w*_*j*_, i.e., $I_{\text{syn}}=\underset{j\in I}{\sum }\underset{t_{j}}{\sum }w_{j}\cdot \delta \left(t-t_{j}\right)$ (see Figure 1Aa).

We consider both deterministic and probabilistic synapses (Branco and Staras, 2009) for which a transmission probability *p*_*r*_ < 1 is tantamount to multiplying presynaptic spikes by the outcome of a Bernoulli trial with probability *p*_*r*_.

#### 2.1.2. Model with leak

In Equation (1) the leak is captured by the term *g*(υ) = −*g*_*L*_(υ − *E*_*L*_) and *I*_syn_ is the sum of conductance-based synaptic inputs. The inputs decay exponentially with time constant τ_syn_ and have a reversal potential *E*_*e*_, that is *I*_syn_ = *g*_*e*_(*t*) · (*V*(*t*) − *E*_*e*_) where:

(2) $$\begin{array}{l} \tau_{\text{syn}}\frac{d}{dt}g_{e}=-g_{e}\left(t\right)+\underset{j\in I}{\sum }\underset{t_{j}}{\sum }w_{j}\cdot \delta \left(t-t_{j}-t_{\text{delay}}\right). \end{array}$$

The sum $\underset{t_{j}}{\sum }\delta \left(t-t_{j}-t_{\text{delay}}\right)$ corresponds to presynaptic spikes of neuron *j* ∈ *I*, each occurring at time *t*_*j*_ and contributing to postsynaptic depolarization in an amount of *w*_*j*_ after a delay *t*_delay_ (see Figure 1Ab).

### 2.2. Synaptic input configuration

We consider the scenario of a single neuron which receives *d* independent synaptic inputs, each associated with a presynaptic neuron (Figure 1Ba). In the model with leak, we model background activity by stimulating the postsynaptic neuron by an external Poisson synaptic current of strength *w*_*n*_ at rate ν (Hô and Destexhe, 2000) (Figure 1Bb).

#### 2.2.1. Model without leak

We assume that presynaptic neurons fire together in short pulses, hereafter termed as “volleys” (Figure 1Ca). In particular, the postsynaptic neuron potential is at rest (i.e., 0) at the start of a volley, and for a volley of duration *T*_*U*_, at time *t*, each presynaptic neuron independently selects a spike uniformly at random in the interval [*t, t* + *T*_*U*_]. The time between two consecutive volleys is *T*_*D*_ ms. Most of our analysis is carried out under the random order assumption that volley spikes are uniformly distributed, although we also consider deviations from this assumption in Section 3.1.2.

#### 2.2.2. Model with leak

Each presynaptic neuron fires according to an inhomogeneous Poisson process (unless differently specified). The rate of the process alternates between DOWN phases of low rate values (λ_*D*_) and UP phases of high rate values (λ_*U*_). The duration of each DOWN and UP phase corresponds to the parameters *T*_*D*_ and *T*_*U*_, respectively (Figure 1Cb). The volleys in the model without leak may be regarded as the limiting case of short UP phases and they lead to analytical tractability of the model. In one setting when we study robustness against input parameter variations we shift the input rate function of each input neuron by some random delay, independently drawn from a uniform distribution in [0, σ].

### 2.3. Learning rule

#### 2.3.1. Memory trace

Each synapse tracks pre- and postsynaptic spikes by means of a scalar memory trace *m*(*t*) which is updated similarly to classical STDP (Morrison et al., 2008). Accordingly, presynaptic spikes that happen in a short interval *T*_early_ before a postsynaptic spike, all increase *m*(*t*) promoting synaptic potentiation. Conversely, presynaptic spikes following a postsynaptic spike in a time window *T*_late_, decrease *m*(*t*) which can lead to synaptic depression (see *green* and *red* shaded regions in Figure 1C). We refer to these spike pair events as potentiation and depression signals and we denote them by the binary indicator variables 𝟙_↑_(*t*) and, respectively, 𝟙_↓_(*t*).

#### 2.3.2. Model without leak

For volley input, these signals simply correspond to the spike order of a synapse in each volley, that is 𝟙_↑_(*t*) is 1 at the time of a postsynaptic spike if it was preceded by a presynaptic spike in that volley and similarly 𝟙_↓_(*t*) is 1 at the time of a presynaptic spike if it was preceded by a postsynaptic spike in that volley.

The memory trace in this setting is given by the moving average of learning signals which are potentiation signals (see Figure 1Da), that is, let *S*^(signals)^(*M*) be a set containing the time of the last *M* learning signals, then

(3) $$\begin{array}{l} m\left(t\right)\equiv M^{-1}\underset{t^{\prime }\in S^{\text{(signals)}}\left(M\right)}{\sum }{𝟙}_{↑}\left(t^{\prime }\right). \end{array}$$

#### 2.3.3. Model with leak

The main difference in this setting is that the neurons can spike more than once in an UP phase instead of at most once in a volley. The memory trace is updated for spikes pairs corresponding to potentiation and depression signals as in the model without leak. However, we restrict the pairs which trigger such signals to those which involve the first postsynaptic spike in an UP phase (*purple* stars in Figure 1). We will hereafter dub such postsynaptic spikes as distinguished spikes (DSPs). This restriction accounts for the fact that STDP may involve more complex interactions between pre- and postsynaptic firing rather than those considered in classic doublet STDP models (Pfister and Gerstner, 2006). To define the memory trace and its update rules formally we therefore first define all relevant variables (for an overview, see Figure 1Db).

For a synapse, we denote by 𝟙_pre_(*t*) and 𝟙_post_(*t*) the indicator variables which are 1 at time *t* if the corresponding pre- or postsynaptic neuron spiked and 0 otherwise. We use these variables to derive indicator variables for potentiation and depression signals, however, for the derivation, we also require variables which monitor whether a DSP recently occurred and whether presynaptic spikes occur in a time window around it. In this regard, we introduce the binary variable *o*_burst_(*t*) which is 1 if a DSP occurred in [*t* − *T*_burst_, *t*), and 0 otherwise. We define it recursively as follows:

(4) $$\begin{array}{l} o_{\text{burst}}\left(t\right)=\underset{t^{\prime }\in S^{\text{post}}\left(t-T_{\text{burst}},t\right)}{\sum }\left(1-o_{\text{burst}}\left(t^{\prime }\right)\right) \end{array}$$

where $S^{\text{post}}\left(t_{1},t_{2}\right)$ denotes the set of all postsynaptic spikes in the interval [*t*_1_, *t*_2_). Similarly, we monitor the depression signal window by the binary variable *o*_late_(*t*) which is 1 in a time window *T*_late_ after a DSP and 0 otherwise, i.e.,

(5) $$\begin{array}{l} o_{\text{late}}\left(t\right)=\underset{t^{\prime }\in S^{\text{post}}\left(t-T_{\text{late}},t\right)}{\sum }\left(1-o_{\text{burst}}\left(t^{\prime }\right)\right). \end{array}$$

Accordingly, the depression and potentiation signal indicator variables are given by

(6) $$\begin{array}{l} {𝟙}_{↓}\left(t\right)={𝟙}_{\text{pre}}\left(t\right)\cdot o_{\text{late}}\left(t\right),\\ {𝟙}_{↑}\left(t\right)={𝟙}_{\text{post}}\left(t\right)\cdot \left(1-o_{\text{burst}}\left(t\right)\right)\cdot \min\left\{1,|S^{\text{pre}}\left(t-T_{\text{early}},t\right)|\right\} \end{array}$$

where $S^{\text{pre}}\left(t_{1},t_{2}\right)$ denotes the set of all presynaptic spikes in the interval [*t*_1_, *t*_2_).

Whenever 𝟙_↓_(*t*) is 1 we update the memory trace *m*(*t*) as follows

(7) $$\begin{array}{l} m\left(t\right)\leftarrow -1+\gamma m\left(t\right) \end{array}$$

where γ defines the attenuation of the pre-existing memory trace. Similarly, whenever 𝟙_↑_(*t*) is 1 we apply

(8) $$\begin{array}{l} m\left(t\right)\leftarrow 1+\gamma m\left(t\right) \end{array}$$

for each presynaptic spike in the interval [*t* − *T*_early_, *t*), i.e., we apply the update $\left|S^{\text{pre}}\left(t_{\text{post}}-T_{\text{early}},t_{\text{post}}\right)\right|$ times.

#### 2.3.4. Weight update

The plasticity of the synapse depends on the value of *m*(*t*). In both the model with and without leak, the update of the synaptic weight is applied similarly. The main difference is that in the model without leak the weight update rule is applied only after every *L*-th update to the memory trace (*L* ≥ *M*) whereas for the model with leak the weight update follows every memory trace update. In a weight update, the weight can change if and only if *m*(*t*) > θ_*P*_ or *m*(*t*) < θ_*D*_, where θ_*P*_ and θ_*D*_ are the depression and potentiation thresholds (the interval [θ_*D*_, θ_*P*_] is shown as a *shaded green* region in Figure 1Db). Formally, the change in synaptic weight is probabilistic, which has been considered before (Standage and Trappenberg, 2006), and, in particular, is subjected to the outcome of a Bernoulli trial with probability *p*_*s*→*w*_ for depression and *p*_*w*→*s*_ for potentiation. This approach is necessary to prevent many synapses from changing their weight together with the risk of destabilizing postsynaptic firing (see Section 3.2.1). The weight update rule is given as follows

(9) $$\begin{array}{c} w(t)\leftarrow w(t)+\{\begin{array}{ll} \Delta_{d}w(t) & \text{with probability }p_{s\to w}\text{ if }m(t)<\theta_{D},\\ \Delta_{p}w(t) & \text{with probability }p_{w\to s}\text{ if }m(t)>\theta_{P},\\ 0 & \text{otherwise}. \end{array} \end{array}$$

With binary synapses, which have weights *w*_*s*_ and *w*_*w*_, the weight changes according to:

(10) $$\begin{array}{l} \Delta_{p}w(t)=\{\begin{array}{ll} w_{s}-w_{w} & \text{if }w(t)=w_{w},\\ 0 & \text{otherwise}. \end{array}\\ \Delta_{d}w(t)=\{\begin{array}{ll} w_{w}-w_{s} & \text{if }w(t)=w_{s},\\ 0 & \text{otherwise}. \end{array} \end{array}$$

### 2.4. Spike latency normalization

In the model with leak, we achieve homeostatic plasticity by normalization of the mean timing of the first postsynaptic spike (that is, the DSP) with respect to an UP phase. In this regard it may be noted that for a given input rate function, the distribution of inter-spike intervals *f*(*t*) of a neuron receiving *d*_*s*_ strong synaptic inputs out of *d* inputs in total is known (Burkitt, 2006a,b), and so is the mean first passage time (Cox and Miller, 1977; Ricciardi, 1977; Tuckwell, 1988). Accordingly, denoting by *t*_DSP_ the relative time of the first postsynaptic spike in an UP phase, and keeping in mind that *f*(*t*) is defined for *t* ∈ [0, *T*_*U*_], the expected first passage time, that is the average relative timing of DSP with respect to the onset of an UP phase is:

(11) $$t_{\text{DSP}}\equiv 𝔼[t_{\text{first}}|t_{\text{first}}<T_{U}]=\int_{t\;=\;0}^{T_{U}}t\cdot f(t)dt.$$

For convenience we define $r\equiv \frac{t_{\text{DSP}}}{T_{U}}$ to be the expected time of the first spike within an UP phase relative to the length of the UP phase. It may then be noted that setting the duration of a DSP (i.e., *T*_burst_) such that *T*_burst_ ≥ *T*_*U*_, ensures that only one DSP occurs in an UP phase. In this fashion, it is possible to distinguish between early vs. late presynaptic spikes, that is input spikes arriving *T*_early_ before and *T*_late_ after the DSP as required by our learning rule (6). Furthermore, choosing *T*_early_, *T*_late_ ≥ *T*_*U*_ enables the contribution of all presynaptic spikes to potentiation/depression in an UP phase (for results related to a large value of *T*_*U*_ see Section 3.2.2). In this fashion, for UP phases sufficiently apart from each other, so as to neglect synaptic changes due to overlapping learning signals from consecutive UP phases, our learning rule performs normalization of the expected spike latency *t*_DSP_. The details of how this is possible are reported in Sections 3.1.1, 3.1.2, and 3.2.1.

### 2.5. Heterogeneous (multimodal) synaptic weights

Besides binary synapses, we also consider multimodal synapses for the model without leak in Section 3.2.3, that is synapses whose weight can assume more than two values. In this regard we explore two updating schemes. The first, additive scheme merely changes synaptic weights by a fixed value ±ω, i.e.,

(12) $$\begin{array}{ll} \Delta_{d}w\left(t\right)=-\omega ,\;\Delta_{p}w\left(t\right)=\omega . & \text{(Additive scheme)} \end{array}$$

The second scheme, which may be regarded as a multiplicative scheme (Van Rossum et al., 2000) instead changes synaptic weights by a randomly rescaled fraction of their value prior to the onset of plasticity, i.e.,

(13) $$\begin{array}{l} \Delta_{d}w\left(t\right)=\left(-c_{d}+\kappa \right)w\left(t\right),\;\Delta_{p}w\left(t\right)=c_{p}+\kappa w\left(t\right)\\ \;\text{(Multiplicative scheme)} \end{array}$$

where *c*_*p*_ and *c*_*d*_ are non-negative constants, κ is a normal distributed random variable with mean 0 and standard deviation ζ > 0, and for both rules *w*(*t*) is set to 0 if negative to ensure *w*(*t*) ≥ 0.

### 2.6. Computational methods

For the model with leak, we use the NEST-simulator (Gewaltig and Diesmann, 2007) (NEST, RRID:SCR_002963) with temporal resolution 0.1 ms and the neuron model iaf_cond_exp, which was introduced by Kumar et al. (2007). For a detailed summary of model parameters and their values used in the simulations, see Tables 1–3.

**Table 1:** **Summary of the model parameters and the default values in the model without leak**.

**Symbol**	**Brief description**	**Value**	**Unit**
*T*_*U*_	Volley length	1	ms
*T*_*D*_	DOWN phase length	1	ms
*d*	Number of neurons in input layer	100	a.u.
τ_ref_	Refractory period	1	ms
θ_*v*_	Firing threshold	10	a.u.
*V*_*r*_	Reset potential	0	a.u.
*T*_burst_	Blocking duration of a distinguished spike (DSP)	1	ms
*T*_early_	Potentiation signal window	1	ms
*T*_late_	Depression signal window	1	ms
*w*_*s*_	Strong weight	1	a.u.
*w*_*w*_	Weak weight	0	a.u.
*p*_*r*_	The probability of successfully transmitting a spike	1	a.u.
*M*	The size of the memory	39	a.u.
*p*_*w*→*s*_	Probability of a weak synapse turning strong	0.05	a.u.
*p*_*s*→*w*_	Probability of a strong synapse turning weak	0.2	a.u.
θ_*D*_	Memory threshold for weight depression	0.26	a.u.
θ_*P*_	Memory threshold for weight potentiation	0.72	a.u.
ω	Additive weight constant	1	a.u.
*c*_*p*_	Potentiation constant in multiplicative weight update	1	a.u.
*c*_*d*_	Depression constant in multiplicative weight update	0.2	a.u.
ζ	Standard deviation of noise in multiplicative weight update	0.15	a.u.

*The parameters θ_D_ and θ_P_, p_w→s_, and p_s→w_ were chosen using the formulas in Section 3.1.1 with ε = 0.5, δ = 0.2*.

**Table 2:** **Summary of the model parameters and the default values in the model with leak**.

**Symbol**	**Brief description**	**Value**	**Unit**
γ	The memory trace decay constant	0.95	a.u.
*T*_*U*_	UP phase length	30	ms
*T*_*D*_	DOWN phase length	50	ms
λ_*U*_	UP phase input rate	40	Hz
λ_*D*_	DOWN phase input rate	0	Hz
*d*	Number of neurons in input layer	100	a.u.
ν	Noise rate	1,000	Hz
*C*	Membrane capacity	250	pF
*E*_*L*_	Leak reversal potential	−70	mV
*g*_*L*_	Leak conductance	16.67	nS
*E*_*e*_	Excitatory reversal potential	0	mV
τ_syn_	Synaptic time constant	0.2	ms
*t*_delay_	Synaptic delay	1	ms
τ_ref_	Refractory period	2.5	ms
*V*_th_	Firing threshold	−55	mV
*V*_*r*_	Reset potential	−60	mV
*T*_burst_	Blocking duration of a distinguished spike (DSP)	35	ms
*T*_early_	Potentiation signal window	35	ms
*T*_late_	Depression signal window	35	ms
*w*_*s*_	Strong weight (in the model with leak)	40	nS
*w*_*w*_	Weak weight (in the model with leak)	1	nS
*w*_*n*_	Noise weight	1	nS

**Table 3:** **Summary of plasticity parameters**.

**Symbol**	**Brief description**	**Unit**	***r* = 1/3**	***r* = 1/2**
*p*_*w*→*s*_	Probability of a weak synapse turning strong	a.u.	0.05	0.05
*p*_*s*→*w*_	Probability of a strong synapse turning weak	a.u.	0.05	0.16
θ_*D*_	Memory threshold for weight depression	a.u.	−14.51	−8.42
θ_*P*_	Memory threshold for weight potentiation	a.u.	1.48	8.70

*The last two columns contain parameter values which fix the relative spike time r for parameters in Table 2*.

## 3. Results

### 3.1. Mathematical analysis

#### 3.1.1. Mechanism of normalization

We start our analysis by deriving some formal results in the model without leak. We restrict our analysis to the scenario of a single neuron which receives *d* excitatory synaptic inputs from presynaptic neurons in a population *I*, of which *d*_*s*_ are strong. We show that there is an equilibrium input of $d_{s}^{*}$ strong synapses such that when the total input weight is ≫ $d_{s}^{*}$, then all synapses have a high chance to decrease their weights. On the other hand, if the total input weight is ≪ $d_{s}^{*}$, then all synapses are more likely to increase their weights. Thus, the equilibrium $d_{s}^{*}$ is stable. The analysis also shows an interval around $d_{s}^{*}$ in which changes of the input weights happen only with very low frequency per volley.

For a single synapse, the total input weight of the postsynaptic neuron determines the expected ratio of potentiation to depression signals. We capture this property by deriving the probability that a synapse receives a potentiation signal instead of a depression signal as a function of the input weight. First, recall that each presynaptic neuron emits exactly one spike per input volley which, in the generic scenario, may be transmitted to the postsynaptic neurons by some probability *p*_*r*_ ≤ 1 (Section 2.1) and, therefore, the postsynaptic neuron spikes at most once per volley. Hence, a potentiation (depression) signal corresponds to a pre/post (post/pre) spike pair within a volley. It should be noted that learning signals are only triggered if both the presynaptic neuron *j* and the postsynaptic neuron spike and, therefore, one needs to condition on a postsynaptic spike. Furthermore, recall from Equation (3) that the memory of the synapse is the moving average of the number of potentiation signals amongst the last *M* learning signals. These signals can be viewed as *M* Bernoulli random variables, where the probability of them being 1 (0) corresponds to the probability of a potentiation (depression) signal. Denote by *X* the number of strong synapses that transmit a spike in a volley. The postsynaptic neuron spikes if *X* ≥ θ_*v*_. For a synapse from the *j*-th input neuron, when *d*_*s*_ out of *d* synapses are strong, *X* is binomially distributed such that *X* ~ *Bi*(*d*_*s*_, *p*_*r*_); accordingly, the probability of the synapse receiving a potentiation signal instead of a depression signal is given by the following expression:

(14) $$\mathrm{Pr}[\text{pot}.\text{ signal}]=\{\begin{array}{ll} p_{{\;}_{\text{early}}}^{\prime }(d_{s})\equiv \frac{1}{\mathrm{Pr}[X\geq \theta_{v}]}\cdot \sum_{i\;=\;\theta_{v}}^{d_{s}}\frac{\theta_{v}}{i\;+\;1}\cdot \mathrm{Pr}[X\;=\;i] & \text{if }w_{j}(t)=w_{w},\\ p_{\text{early}}(d_{s})\equiv \frac{1}{\mathrm{Pr}[X\geq \theta_{v}]}\cdot \sum_{i\;=\;\theta_{v}}^{d_{s}}\frac{\theta_{v}}{i}\cdot \mathrm{Pr}[X\;=\;i] & \text{if }w_{j}(t)=w_{\text{s}}. \end{array}$$

**Figure 2:**
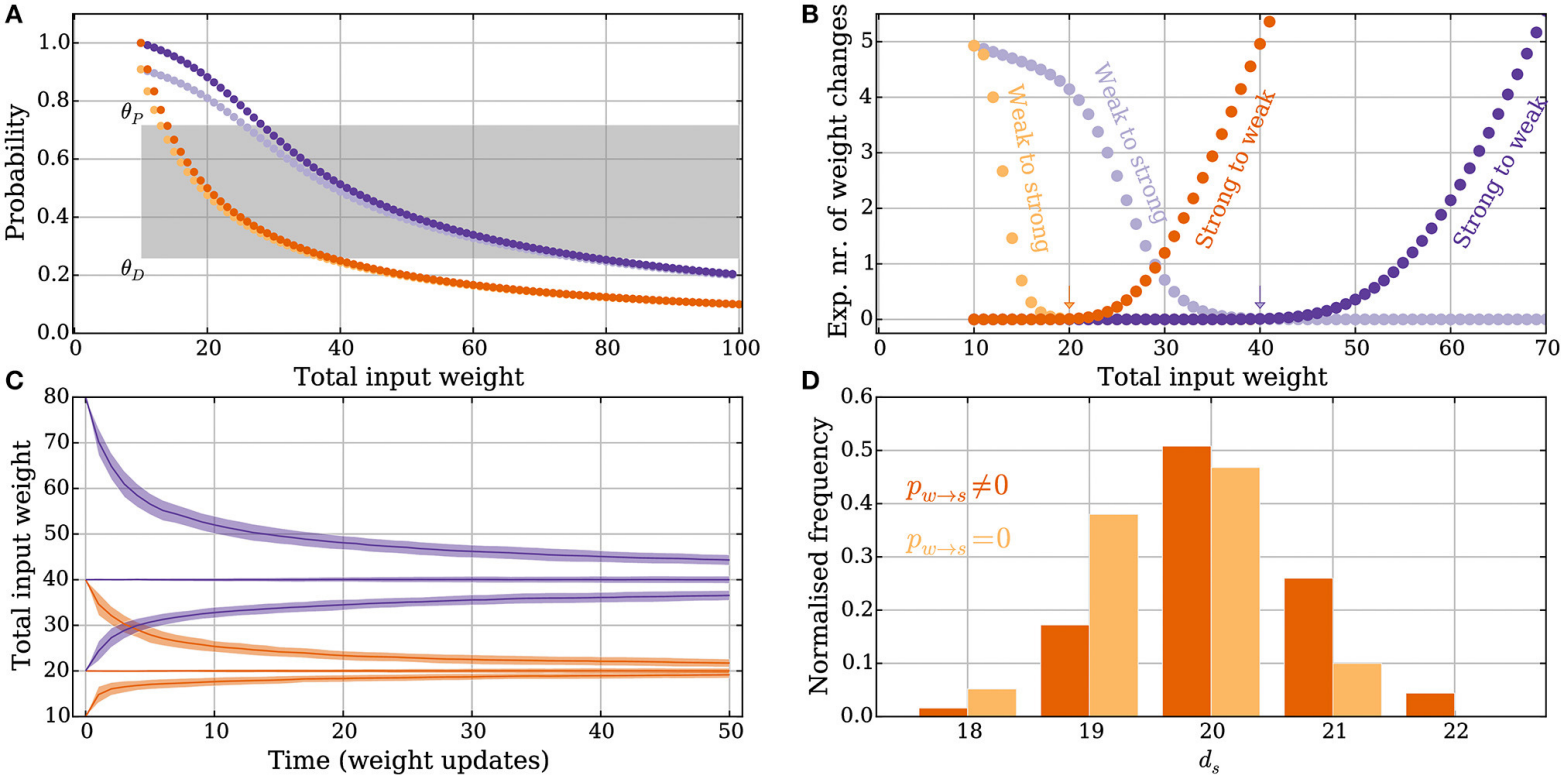
**Principles of the intrinsic homeostasis mechanism in the model without leak**. *Orange curves* correspond to a setting with “deterministic” synapses, which reliably transmit spikes, and the *purple curves* correspond to a setting with “probabilistic” synapses that transmit spikes with probability *p*_*r*_ = 0.5. **(A)** Probability of a potentiation signal conditioned on the postsynaptic neuron spiking and the synapse being reliable. The *gray area* corresponds to the value of the memory trace where synapses neither potentiate nor depress. **(B)** Expected drift toward the stable input weight in each weight update as a function of the total input weight. The *arrows* correspond to the stable input weights to which the input weight converges in the two settings (the stable state). **(C)** Convergence to the stable input weight for three different starting conditions in each setting, where one is the stable state. The *curves* represent the mean over 50 trials and the envelope corresponds to a standard deviation estimate. **(D)** The total input weight, which is the same as the number of strong synapses *d*_*s*_, distribution for deterministic synapses after applying 200 weight updates (*darker orange*) when *d*_*s*_ = 30 before the first weight update. The total input weight distribution is similar to a setting where potentiation is forbidden (*p*_*w*→*s*_ = 0, *lighter orange*), which illustrates that the negative feedback does not undershoot the stable state by much. For all panels, *M*, θ_*D*_ and θ_*P*_, *p*_*w*→*s*_, and *p*_*s*→*w*_ are set as in Table 1.

It thus follows that if the pre- or postsynaptic neuron do not spike in a volley, the memory of the synapse does not change. Hence, the probability of a depression signal is 1−Pr[pot. signal]. The expressions in Equation (14) are shown in Figure 2A for deterministic synapses (*p*_*r*_ = 1, *orange*) and probabilistic synapses (*p*_*r*_ = 0.5, *purple*). The small difference between a weak and a strong synapse is caused by the factor $\frac{\theta_{v}}{i}$ for strong synapses and $\frac{\theta_{v}}{i+1}$ for weak ones. For strong synapses this factor corresponds to being amongst the first θ_*v*_ input signals out of *i* strong input signals whereas for weak synapses this factor corresponds to the signal arriving before any of the first θ_*v*_ strong input signals where the number of possible places in the order is *i* + 1.

The memory trace *m*(*t*) from Equation (3) is an unbiased estimator of Pr[pot. signal] from Equation (14). Furthermore, the probability depends in a monotone fashion on *d*_*s*_ (Figure 2A). This monotone dependence is the principle behind the intrinsic homeostasis effect of the learning rule which allows a synapse to estimate if the total input weight toward the target neuron is too large or too small by just observing and keeping track of recent spike pair orders within a volley. This mechanism results in a stabilizing effect on *d*_*s*_ that we quantify below.

In the model without leak, recall that we apply the weight update rule after every *L*-th volley. For deterministic synapses, we set *L* = *M*. This assumption makes the weight updates independent and, thus, greatly simplifies analysis (see Appendix in Supplementary Material). In the setting with probabilistic synapses, this property holds with high probability by choosing *L* a bit larger. There, we set $L=\frac{2M}{p_{r}\cdot \mathrm{Pr}\left[X\geq \theta_{v}\right]}$ where, as above, *X* is the number of synapses that transmit a spike. *L* depends on *d*_*s*_ for probabilistic synapses because the target neuron cannot be reliably activated if *d*_*s*_ is small.

Denote by Δ_*s*→*w*_(*d*_*s*_) (Δ_*w*→*s*_(*d*_*s*_)) the number of strong (weak) synapses that turn weak (strong) after applying the weight update rule. Recall that the weights are binary 0 and 1 in the model without leak, so the number of strong synapses is the same as the total input weight. The expected number of weight changes of each type is given by

(15) $$𝔼[\Delta_{s\to w}(d_{s})]=\mathrm{Pr}[Bi(d_{s},p_{\text{early}}(d_{s}))\leq \theta_{D}M]\cdot d_{s}\cdot p_{s\to w},$$

(16) $$\begin{array}{l} 𝔼[\Delta_{w\to s}(d_{s})]=\mathrm{Pr}[Bi(d-d_{s},p_{\text{early}}^{\prime }(d_{s}))\geq \theta_{P}M]\\ \;\cdot (d-d_{s})\cdot p_{w\to s}. \end{array}$$

The expected number of weight changes of each type is shown in Figure 2B for deterministic (*orange*) and probabilistic synapses (*p*_*r*_ = 0.5, *purple*). The total input weight change (or “weight drift”) after a learning step is given by

(17) $$\Delta_{w\to s}(d_{s})-\Delta_{s\to w}(d_{s}).$$

For ε > 0, the expected number of weight changes Δ_*w*→*s*_(*d*_*s*_) + Δ_*s*→*w*_(*d*_*s*_) for a fixed number of strong input synapses can be bounded by choosing

(18) $$\theta_{D}=(1-\varepsilon )\cdot p_{\text{early}}(d_{s})\text{ and }\theta_{P}=(1+\varepsilon )\cdot p_{\text{early}}^{\prime }(d_{s}),$$

and for some δ > 0 by choosing

(19) $$M=\left\lceil \frac{3\log(\delta^{-1})}{\varepsilon^{2}p_{\text{early}}^{\prime }(d_{s})}\right\rceil .$$

A detailed derivation of Equation (19) may be found in the Appendix (Section A2 in Supplementary Material). For this choice of *d*_*s*_, the expected fraction of synapses that change their weight is less than δ; i.e., 𝔼[Δ_*w*→*s*_(*d*_*s*_) + Δ_*s*→*w*_(*d*_*s*_)] ≤ δ*d*. It may be noted that this choice of *M* is independent of the parameters *p*_*s*→*w*_ and *p*_*w*→*s*_. Their roles in the learning rule are simply to reduce the weight drift. Details on how to choose them are given in Section 3.1.2.

In the following, we argue that in the model with leak, the memory trace is a biased estimate of the expected relative spike time *r* of a DSP within an UP phase. Because of this feature we will refer to the learning rule as the spike latency normalization (SLN) rule. Assume we are in a static setting where the weights are fixed such that the expected relative spike time is *r*. Let *Y*_*i*_ denote the result of the *i*-th learning signal: 1 if it was a potentiation signal and 0 otherwise. By ignoring the effect of a specific input neuron on the spike time of the post neuron (which is valid if θ_*v*_ is large with respect to *w*_*s*_) and by assuming that the input neuron spikes only once in the interval [0, *T*_*U*_] (which is valid if *T*_*U*_ is small compared with 1/λ_*U*_), a synapse expects a potentiation signal with probability *r*. That is, the variables *Y*_*i*_ can be assumed to be Bernoulli random variables with parameter *r*. The expected value of the memory trace after ℓ learning signals depends linearly on *r* and is given by

(20) $$\begin{array}{l} 𝔼\left[m\left(t\right)\right]=\overset{\ell -1}{\underset{i\;=\;0}{\sum }}𝔼\left[\gamma^{i}\left(2Y_{i}-1\right)\right]=\overset{\ell -1}{\underset{i\;=\;0}{\sum }}\gamma^{i}\left(2r-1\right)\overset{\ell \to \infty }{\underset{\;}{\to }}\frac{2r-1}{1-\gamma }. \end{array}$$

Since the trials are independent, the variance of the distribution can also be easily computed and equals

(21) $$\begin{array}{l} \text{Var}\left[m\left(t\right)\right]=\overset{\ell -1}{\underset{i\;=\;0}{\sum }}\text{Var}\left[\gamma^{i}\left(2Y_{i}-1\right)\right]\\ \;=\overset{\ell -1}{\underset{i\;=\;0}{\sum }}\gamma^{2i}4r\left(1-r\right)\overset{\ell \to \infty }{\underset{\;}{\to }}\frac{4r\left(1-r\right)}{1-\gamma^{2}}. \end{array}$$

An example of the memory trace distribution is presented in Section 3.2. Furthermore, in Section 3.2.5 we show the expected value of the memory trace 𝔼[*m*(*t*)] as a function of *d*_*s*_ for a fixed UP phase length *T*_*U*_. 𝔼[*m*(*t*)] decreases monotonously as a function of *d*_*s*_. This monotone dependence is the principle behind the intrinsic homeostasis mechanism just as *p*_early_ is in the model without leak.

#### 3.1.2. Convergence, fast homeostasis, and the random spike order assumption

In the model without leak, our rule converges to a value $d_{s}^{*}$ of the total input weight that represents the stable state (or attractor) of the learning process. This state minimizes the expected learning error, which may be quantified by the absolute value of the mean weight drift, that is

(22) $$\begin{array}{l} d_{s}^{*}\equiv \underset{d_{s}}{\mathrm{argmin}}𝔼\left[\left|\Delta_{w\to s}\left(d_{s}\right)-\Delta_{s\to w}\left(d_{s}\right)\right|\right]. \end{array}$$

The stable state is shown with an arrow in Figure 2B. Equations (15) and (16) show that the stable state depends on the parameters θ_*P*_, θ_*D*_, *p*_*s*→*w*_, and *p*_*w*→*s*_.

In the previous section, *M*, θ_*P*_, and θ_*D*_ were chosen to enforce low expected weight change in some state *d*_*s*_. We will apply these choices to the steady state $d_{s}^{*}$. Observe that since 𝔼[Δ_*w*→*s*_(*d*_*s*_)] is a decreasing function and 𝔼[Δ_*s*→*w*_(*d*_*s*_)] is an increasing function (Figure 2B) we can scale both expectations independently by changing *p*_*w*→*s*_ and *p*_*s*→*w*_, respectively. In this way, $d_{s}^{*}$ can be adapted.

Starting with $d_{s}>\left(1+2\varepsilon \right)d_{s}^{*}$, it may be shown that (Appendix A3 in Supplementary Material) the expected number of weight updates *T* applied, until $d_{s}<\left(1+2\varepsilon \right)d_{s}^{*}$ is upper bounded as follows

(23) $$𝔼[T]\leq \frac{1}{(1-2\varepsilon )\cdot p_{s\to w}}\cdot \left(\frac{1}{d_{s}^{*}}+\log(d_{s}-(1+2\varepsilon )d_{s}^{*})\right).$$

That is, the average number of weight updates is logarithmic in the distance from the target value and inversely proportional to *p*_*s*→*w*_, which is the probability of a strong synapse turning weak when *m*(*t*) < θ_*D*_. To avoid dangerous oscillations on synaptic weights however, this latter probability should be small so as to prevent the total input weights from dropping to 0. A similar consideration, could also be made for *p*_*w*→*s*_ insofar as a large value of this probability could result in an overshooting (i.e., strong global potentiation) of synaptic weights, with the important difference however, that, in our formulation, strong potentiation still allows the postsynaptic neuron to fire, whereas strong depression would ultimately result in the shutdown of synaptic inputs. In this catastrophic scenario, the postsynaptic neuron would not be able to fire further, and would become disconnected.

To avoid this possibility and unnecessary weight fluctuations, we present a heuristic criterion to choose *p*_*s*→*w*_ and *p*_*w*→*s*_ which allow to upper bound the expected weight change by $\left\lceil \varepsilon_{1}d_{s}^{*}\right\rceil$ if $ds\in [(1-2\varepsilon_{1})d_{s}^{*},(1+2\varepsilon_{1})d_{s}^{*}]$ for some ε_1_ > 0. For $d_{s}=(1+2\varepsilon_{1})d_{s}^{*}$ the expected number of synapses that depress when a weight update is applied is given by $𝔼[\Delta_{s\to w}((1-2\varepsilon_{1})d_{s}^{*})]$ as in Equation (16). Similarly, the number of synapses that potentiate in when a weight update is applied is given by $𝔼[\Delta_{s\to w}((1-2\varepsilon_{1})d_{s}^{*})]$ as in Equation (15). To prevent undershooting when $d_{s}>(1+2\varepsilon_{1})d_{s}^{*}$, it suffices to choose $p_{s\to w}<\frac{c^{\prime }(d_{s}^{*}-\theta_{v})}{d}$ for a sufficiently small *c*′ (e.g., *c*′ = 2 in this study). The above conditions translate to setting the model parameters such that:

(24) $$p_{s\to w}=\min\left(\frac{c^{\prime }(d_{s}^{*}-\theta_{v})}{d},\frac{\varepsilon_{1}d_{s}^{*}}{𝔼[\Delta_{w\to s}((1+2\varepsilon_{1})d_{s}^{*})]}\right)$$

(25) $$\begin{array}{l} p_{w\to s}=\frac{\varepsilon_{1}d_{s}^{*}}{𝔼\left[\Delta_{s\to w}\left(\left(1-2\varepsilon_{1}\right)d_{s}^{*}\right)\right]}. \end{array}$$

Convergence to a stable state where *p*_*s*→*w*_ and *p*_*w*→*s*_ are set as in Equations (24) and (25) is shown in Figure 2C, for both deterministic (*orange*) and probabilistic (*purple*) synapses in the model without leak. The distribution of the total input weight at equilibrium is shown for the deterministic synapses in Figure 2D, where it is compared with a setting where potentiation is turned off, i.e., where the rule only provides negative feedback. The result in Figure 2D shows that if negative feedback is the only requirement, then it suffices to use the memory trace exclusively for weight depression. If the input to the neuron increases too much, e.g., through some other form of short-term Hebbian learning, then the negative feedback provided by the memory mechanism of the strong synapses can quickly reduce the input weight back to a stable state without undershooting it. In both settings described above, the initial total input weight was *d*_*s*_ = 30 and the distribution in the figures is shown after 200 weight updates have been applied.

**Figure 3:**
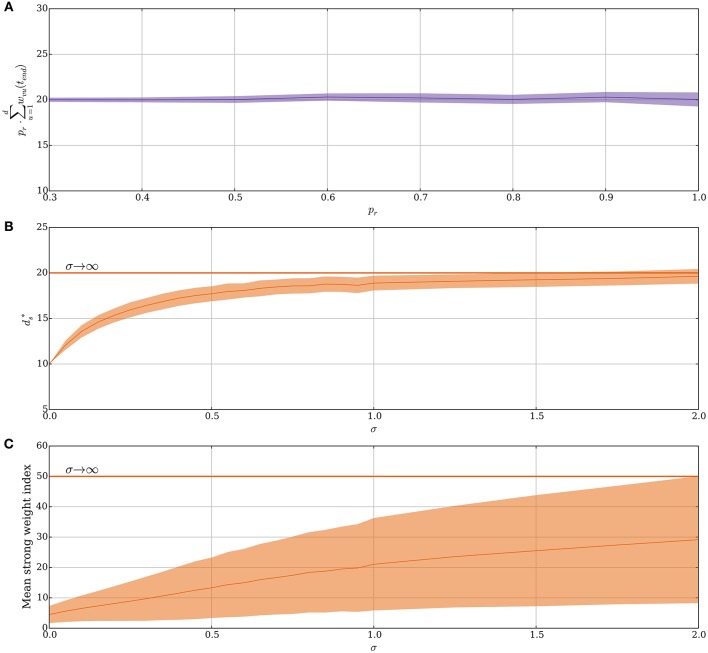
**Noisy input and the random order assumption in the model without leak (Section 3.1.2). (A)** The stable state $d_{s}^{*}$ scales by 1/*p*_*r*_ for probabilistic synapses. The initial value of *d*_*s*_ is set to 20/*p*_*r*_ for each *p*_*r*_ and the plot shows the mean value of *d*_*s*_ · *p*_*r*_ after 50 weight updates over 100 trials. **(B–C)** From a fixed order to a random order. Spike order distributions that are not uniform are considered in these panels. The parameter σ brings them from a fixed order (σ = 0) to a uniform order (σ → ∞). (details are explained in Section 3.1.2). Initially, each synapse is strong independently, with probability 4θ_*v*_/*d*. **(B)** Small variability brings the stable state $d_{s}^{*}$ close to same state as when the spike order is uniform. **(C)** The plasticity mechanism prefers early neurons when σ is small. This is reflected by the mean index of the strong synapses, which are indexed from 1 to *d* in ascending order of expected spike time. However, as σ increases, the early neurons become less distinguishable from later neurons. In **(B,C)** each data point is the mean of 250 trials, where for each trial 250 weight updates are simulated. For all panels, the plasticity parameters are chosen as in Table 1 and the envelopes represent standard deviation estimates.

Figure 3A shows how the stable state $d_{s}^{*}$ is affected by probabilistic synapses. In particular, in this case $d_{s}^{*}$ increases by a factor of $p_{r}^{-1}$ if other parameters remain fixed. The intuition behind this relation comes from two observations. First, for that state, the expected total input weight is the stable state for deterministic synapses. Second, if the number of strong synapses *d*_*s*_ is large enough, then the total input *X* = *Bi*(*d*_*s*_, *p*_*r*_) is concentrated around its mean μ_*X*_ = *d*_*s*_*p*_*r*_ with standard deviation $\sigma_{X}=\sqrt{d_{s}p_{r}\left(1-p_{r}\right)}$. Asymptotically for growing θ_*v*_, *d*_*s*_, and *d*, the main contribution in the sum in Equation (14) comes from terms close to the mean of the form

(26) $$\begin{array}{l} \frac{\theta_{v}}{\mu_{X}\pm \Theta \left(\sigma_{X}\right)}=\frac{\theta_{v}}{\mu_{X}\left(1\pm \Theta \left(\sigma_{X}^{-1}\right)\right)}=\left(1\mp \Theta \left(\sigma_{X}^{-1}\right)\right)\cdot \frac{\theta_{v}}{\mu_{X}}\\ \;=\left(1\mp \Theta \left({d_{s}}^{-1/2}\right)\right)\cdot \frac{\theta_{v}}{d_{s}p_{r}}. \end{array}$$

In Equation (26) above, the notation *f* = Θ(*g*) means that *f* is bounded by *g* both above and below asymptotically.

Our hitherto analysis has considered the input order to be uniformly distributed (i.e., “random order” assumption), yet one may also ask how the SLN rule performs if this assumption is relaxed. Indeed, spikes are known to have a precise repeating temporal structure in some cortical areas related to sensory processing (Bair and Koch, 1996; Heil, 1997; Fellous et al., 2004) and standard STDP is known to tune to the first spike for such repeating spike patterns (Guyonneau et al., 2005). To model the transition from an orderly spike distribution to a uniform one the expected relative spike time of every input neuron in a volley is fixed but the deviation of the spike time is varied. Formally, for the *i*-th neuron a normal random variable $Z_{i}\sim N\left(i/d,\sigma_{Z}\right)$ is sampled. These variables impose an order on the input spikes within a volley in a natural way. For the resulting order, the input neurons spike in an equi-spaced manner. This approach is valid since we ignore membrane leakage and, therefore, the spike order is the only temporal structure that has any influence on the distribution of potentiation and depression signals. For σ_*Z*_ = 0, the ordering is fixed as 1, 2, …, *d* and for a growing σ_*Z*_, the ordering approaches a uniform distribution. Figure 3B shows how this choice of input spike distribution affects the stable state $d_{s}^{*}$. Not surprisingly, if the order is fixed (σ_*Z*_ = 0), then only the first θ_*v*_ synapses get strengthened as was the case in Guyonneau et al. (2005) where classic STDP by a repeating spike pattern turned the neuron into a detector for the start of the pattern. However, already a small value of σ_*Z*_ quickly shifts the stable state toward the same one as for a random input order. For σ_*Z*_ = 2.0 the first 20 input neurons are close to a random permutation since the probability of the 20th neuron being earlier than the first neuron is approximately 0.47. Figure 3C shows the mean index of strong synapses. Even though the stable state remains close to the same for small values of σ_*Z*_, the early synapses are the ones that are preferably strengthened in agreement with Guyonneau et al. (2005). For other choices of spike order distributions, the negative feedback of the intrinsic homeostasis mechanism still applies (Appendix A4 in Supplementary Material).

### 3.2. Numerical simulations

#### 3.2.1. Stability in the model with leak

**Figure 4:**
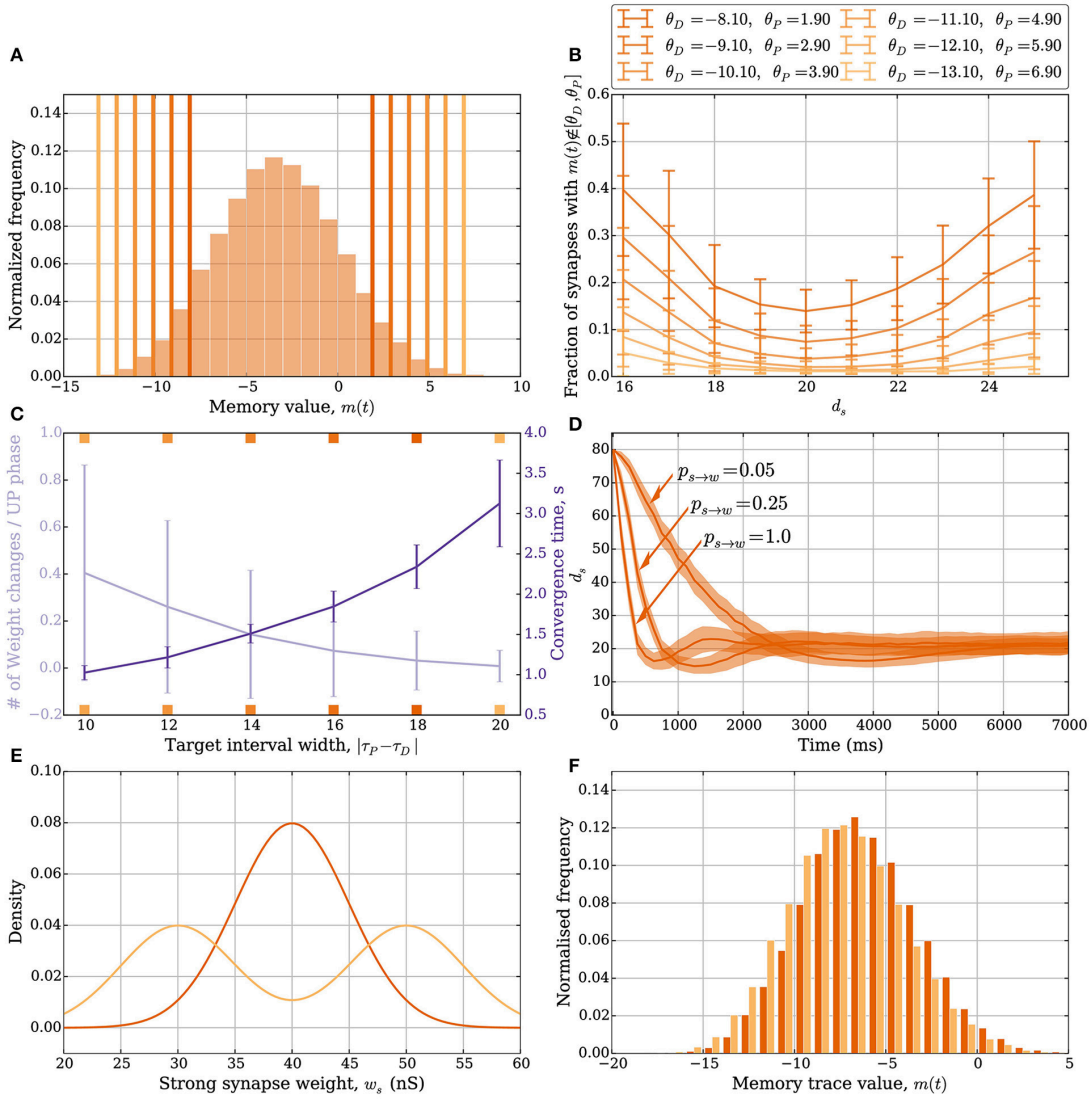
**Plasticity vs. stability in the model with leak (Section 3.2.1). (A)** Distribution of the memory trace in a static setting, with *p*_*s*→*w*_ = *p*_*w*→*s*_ = 0 and *d*_*s*_ = 20 strong input synapses. The *colored vertical bars* represent the target interval [θ_*D*_, θ_*P*_] used in **(B,C)**. **(B)** The mean fraction of synapses whose memory trace lies outside the interval [θ_*D*_, θ_*P*_] in a static setting. **(C)** The trade-off between the number of weight changes in the stable state and convergence time to the stable state. The *light purple curve* shows the mean number of weight changes per UP phase over all input synapses. The *dark purple curve* shows the mean time for the weights to converge from *d*_*s*_ = 80 to *d*_*s*_ = 25. The *ticks* on the x-axis are colored and correspond to the thresholds in **(A)** and *curves* in **(B)**. In this setup, the number of strong synapses in the stable state is *d*_*s*_ = 20. This is achieved by choosing *p*_*s*→*w*_ and *p*_*w*→*s*_ such that they satisfy the equation *d*_*s*_*p*_*s*→*w*_ = (*d* − *d*_*s*_)*p*_*w*→*s*_; i.e., *p*_*w*→*s*_ = 0.0625 and *p*_*s*→*w*_ = 0.25. The error bars represent standard deviation estimates. **(D)** Comparison of the effect *p*_*s*→*w*_ and *p*_*w*→*s*_ have on convergence. In this figure, θ_*D*_ = −5.1 and θ_*P*_ = −1.1, and three different values of *p*_*w*→*s*_ (0.05, 0.25, and 1.0) are compared (the corresponding *p*_*s*→*w*_ values are 0.01, 0.06, and 0.25). If too many synapses change their weight simultaneously because of a small target interval and large weight change probabilities, then the weights can overshoot the stable state and oscillate around it. **(E,F)** Stability for different weight distributions. **(E)** Density function for two different weight distributions of the strong synapses. The *dark orange* is the density function of N(40,5), and the *light orange* is the density function for the random experiment, where we either draw the weight from the distribution N(35,5) or N(45,5) based on a fair coin toss. **(F)** The corresponding memory trace distribution for each weight distribution when *d*_*s*_ = 24 in a static setting with *p*_*s*→*w*_ = *p*_*w*→*s*_ = 0. By choosing θ_*D*_ = −16 and θ_*P*_ = 5, the weight remains unchanged for both weight distributions over a period of 50 s and oscillating input (data not shown). In **(A)** the distribution is obtained by sampling memory traces of all *d* synapses after a simulation of 500 UP phases in 250 trials. In **(B)** each data point is the mean of 100 trials. In **(C)** each curve is the average over 40 trials. In **(D)** each curve is the average over 200 trials with a data point every 125 ms. For all panels, error bars and envelopes represent standard deviation estimates. In **(F)** the distribution is sampled from 100 synapses over 200 trials of a 50 second simulation.

As in the model without leak, the number of weight changes in the model with leak is mainly determined by two factors: (i) the number of synapses whose memory trace lies outside the target interval [θ_*D*_, θ_*P*_] and (ii) the weight update parameters *p*_*s*→*w*_ and *p*_*w*→*s*_. How the width of the interval [θ_*D*_, θ_*P*_] affects plasticity is shown in Figures 4A–C, whereas an example of how *p*_*s*→*w*_ and *p*_*w*→*s*_ can affect plasticity is shown in Figure 4D. The width of the target interval [θ_*D*_, θ_*P*_] determines how many synapses can change their weight in the stable state. Such intervals are laid over the memory trace distribution in Figure 4A, which was sampled by fixing *d*_*s*_ = 20. The tails of the distribution outside the interval correspond to the synapses that can change their weight. Since these tails fall off exponentially if the learning signals are independent Bernoulli trials, the fraction of synapses that can update their weights in the stable state can be made arbitrarily small by choosing a large interval. It may be noted that the parameter γ also needs to be chosen sufficiently close to 1, which corresponds to choosing *M* larger in the model without leak. Therefore, one can choose parameters such that a region around the stable state induces almost no weight changes, thus avoiding unnecessary fluctuations of total input weight. However, choosing a large interval also leads to diminished plasticity in regions further away from the stable state (Figure 4B). This suggests that a trade-off exists between synaptic weight plasticity and the time to reach close to the stable state.

Figure 4C shows such trade-off where the *orange* curve represents the expected number of weight updates per UP phase when *d*_*s*_ = 20 (over all synapses) whereas the *purple* curve shows the mean time to remain with 25 strong synapses starting from 80. For Figures 4A–C, the parameters *p*_*s*→*w*_ and *p*_*w*→*s*_ were set to 0.25. In combination with a small target interval, a bad choice of *p*_*s*→*w*_ and *p*_*w*→*s*_ can lead to plasticity over- or undershooting as in the model without leak (see Section 3.1.2). Undershooting the input weight in the stable state is a concern if it results in the weights being too small to activate the target neuron. Overshooting is less of a concern for activation, but it is wasteful since fewer weight updates can be used to reach the stable state. An example of undershooting is shown in Figure 4D, where θ_*P*_ = −1.1 and θ_*D*_ = −5.1. Over- and undershooting happen in this setting because the memory trace depends on learning signals from the past that are irrelevant when the weights are close to converging. To handle this effect, weight updates can be limited by choosing *p*_*s*→*w*_ and *p*_*w*→*s*_ small enough. Furthermore, since input neurons in the model with leak do not always spike in an UP phase only a fraction of the input synapses update their memory trace and trigger the weight update rule. This results in a natural tie-breaking mechanism that prevents all synapses from updating their weights simultaneously.

The SLN rule achieves normalization quickly. Though speed is a desirable property (see the work of Zenke et al., 2013 for a discussion on the necessity of a fast-homeostatic mechanism), it is also important to be energy efficient; that is, the total number of synaptic weight changes should be small. Different synaptic weight distributions can result in the same value of expected relative postsynaptic spike time. A feature of stability in the SLN rule is that this does not result in further weight changes. We highlight these features in Figures 4E,F. Figure 4E shows two different synaptic weight distributions for the strong weight synapses. One is normally distributed with mean 40 nS and standard deviation 5 nS (*dark orange*). The other is obtained by sampling from a normal distribution as well, with the same standard deviation, by first flipping a fair coin to decide if the mean should be 35 or 45 nS (*light orange*). The resulting distribution of the memory trace is shown in Figure 4F when *p*_*s*→*w*_ = *p*_*w*→*s*_ = 0 and *d*_*s*_ = 24. By setting θ_*D*_ = −16 and θ_*P*_ = 5, the synaptic weights remain unchanged over 1 min of input activity (data not shown). That is, both weight distributions are stable.

#### 3.2.2. Short vs. long UP phases

**Figure 5:**
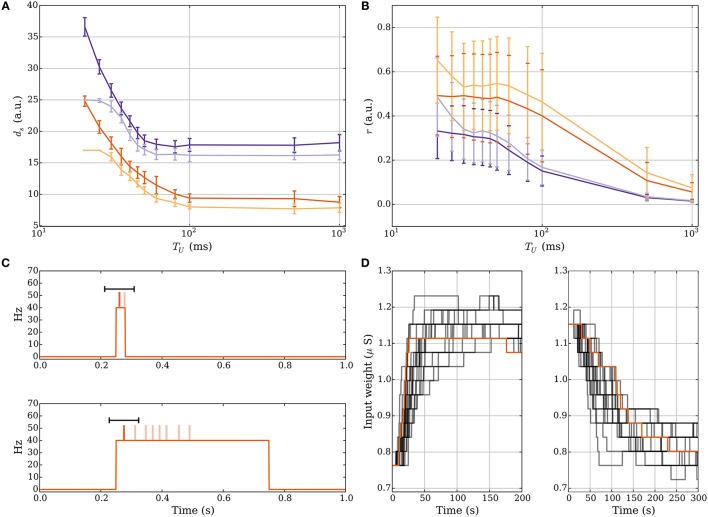
**Comparison of short and long UP phases. (A,B)** Dependence of number of strong input synapses *d*_*s*_ and relative spike time on the lengths of UP phases. We set *T*_*D*_ = 1000.0 ms and we set *T*_burst_ = 1000.0 such that there can be only one DSP per UP phase. **(A)** shows that for long UP phases the mechanism normalizes *d*_*s*_. **(B)** shows that for short UP phases the mechanism normalizes the relative spike time *r* within an UP phase. **(C)** Depression is stronger for sufficiently long UP phases. For two different rate functions the postsynaptic spikes are drawn on top of an UP phase where darker spikes represent DSPs. The *black* bar represents the learning windows. The time window of the depression signal is completely within the UP phase if the UP phase is long. This increases the expected number of depression signals. To compensate, the input weight needs to decrease in order to correct the ratio of potentiation to depression signals. **(D)** Weight change for short UP phases of 30 ms with *d*_*s*_ = 17 at *t* = 0 (left panel) and long UP phases of 0.5 s with *d*_*s*_ = 27 at *t* = 0 (right panel). We show 20 example runs with one highlighted in *orange* for clarity.

For short UP phases the SLN rule normalizes the expected relative spike time *r* of the postsynaptic neuron within an UP phase whereas for long UP phases the rule normalizes the absolute expected latency of the postsynaptic neuron, which is equivalent to normalizing the total input weight in this setting. Figures 5A,B demonstrate how the number of strong input synapses *d*_*s*_ and the relative spike time *r* change when varying the length of the UP phase if we start with *d*_*s*_ = 25 and run the process until the weights converge. We use a log-scale for the x-axis to show data for very long UP phases. Figure 5A shows that for long UP phases the input weight is normalized whereas Figure 5B shows that for short UP phases the relative spike time is normalized. We show UP phases of length 20.0, 25.0, 30.0, 35.0, 40.0, 45.0, 50.0, 60.0, 80.0, 100.0, 500.0, and 1000.0 ms. The length of the down phase is set to 1000.0 ms and we set *T*_burst_ = 1000.0 such that there can be only one DSP per UP phase. We use the plasticity parameters in Table 3 for *r* = 1/3 (*dark purple*) and *r* = 1/2 (*dark orange*) where the lighter colored curves correspond to a setting with *p*_*w*→*s*_ = 0.0 as in Section 3.1.2. The schematic in Figure 5C explains the reason for this difference. The figure shows two different rate functions with short (30 ms) and long (500 ms) UP phases. Postsynaptic spikes are drawn on top of the rate function for reference where darker spikes represent DSPs. The windows for potentiation and depression signals are drawn in *black* over the DSPs. For short UP phases the windows for potentiation and depression signals cover the whole UP phase. In this setting, the fraction of potentiation signals represents the relative spike time within an UP phase. In contrast, for long UP phases, the window of depression lies completely within the UP phase. To compensate for more depression signals the input weight needs to decrease such that the ratio of potentiation to depression signals is within the target regime. Figure 5D shows the weight over time for short UP phases of 30 ms with *d*_*s*_ = 17 at *t* = 0 (*left panel*) and long UP phases of 0.5 s with *d*_*s*_ = 27 at *t* = 0 (*right panel*). In both panels the DOWN phases are of length 1 s. We use the plasticity parameters for *r* = 1/3 in Table 3 which corresponds to the *dark purple* curve in panels A-B. Each timescale captures 400 UP phases and we show 20 example runs with one highlighted in *orange* for clarity.

For long UP phases we chose *T*_burst_ sufficiently large to permit only one DSP per UP phase. However, for short DOWN phases this leads to a DSP blocking first spikes of subsequent UP phases becoming DSPs. As an alternative, one can assume that a DSP is only triggered if the membrane potential was recently in a low state of activity and a DSP blocks further DSPs until a low activity state is reached again. One can further assume that spike pairs are only considered to trigger learning signals if the subthreshold activity remains above some threshold between both spikes. This voltage based approach fixes the issue above with short DOWN phases and it implies that for constant or high rate input (as we study in Section 3.2.6), where the subthreshold potentiation remains large, neither the memory nor, hence, the synaptic weight, would change.

#### 3.2.3. Heterogeneous/multimodal weights

**Figure 6:**
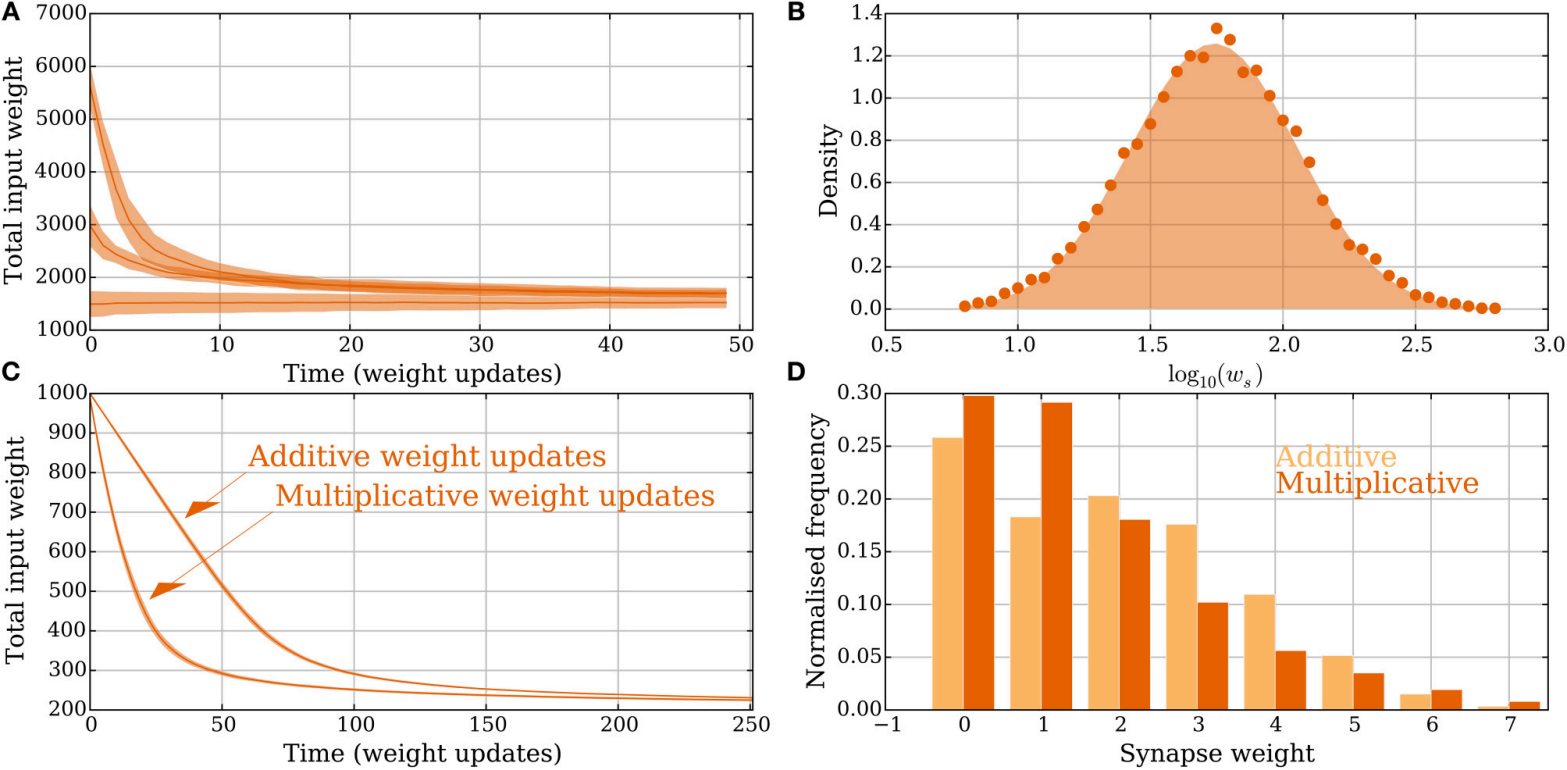
**Heterogeneous and multimodal weights in the model without leak (Section 3.2.3). (A,B)** Heterogeneous weights. **(A)** Input convergence with the spike threshold θ_*v*_ adapted to the synaptic weights. **(B)** Comparison between the distribution of strong synaptic weights (lognormal distribution, *shaded*) and the strong weights after convergence (*dots*). **(C,D)** Multimodal synapses. Two different weight update rules are presented: one additive and one multiplicative (see Section 2.5 for details). **(C)** Convergence for the two different setups where all the synapses start with weight 10 and θ_*v*_ is set to 100. **(D)** The input weight distributions are compared for the multimodal weight update rules in **(C)** after 250 extra weight updates. The additive weight update rule produces a bimodal weight distribution whereas the multiplicative one produces a unimodal distribution. None of the update rules imposes a hard upper bound on the weights and all resulting weights are less than 10, which was the starting weight of the synapses. For all panels, the parameters were chosen as in Table 1 unless otherwise specified. In **(A,C)** each curve is the mean of 30 and, respectively, 100 trials; the envelopes represent a standard deviation estimate. In **(B)** the distribution is obtained from 5.000 trials, where in each one 50 weight updates were simulated.

The SLN rule also works for heterogeneous and multimodal synapses although the exposition so far was restricted to binary synapses. This more general scenario is discussed in the model without leak of mere SIF neurons, with results from simulations presented in Figure 6.

In the setting with heterogeneous weights *w*_*w*_ = 0 for all synapses, whereas *w*_*s*_ is drawn independently for each synapse from a distribution as in Barbour et al. (2007). The distribution is the one reported in Loewenstein et al. (2011), where $w_{s}=e^{N\left(\mu ,\sigma \right)}$ with μ = 1.74 and σ^2^ = 0.1002. The mean of the distribution is 10^μ+σ^2^/2^ ≈ 5.726. To compare this with the binary weight setting where θ_*v*_ = 10, the threshold is set to $\theta_{v}=10\cdot e^{\mu +\sigma^{2}/2}$. Convergence to a stable state is shown in Figure 6A. Initially 20, 40, or 80 synapses are strong. After convergence, the weight distribution of synapses selected to be strong is compared with the lognormal distribution from above (Figure 6B). The empirical distribution is composed of 5.000 trials and the neuron and synapse parameters (except θ_*v*_ and the weight) are in Table 1.

Figures 6C,D compare convergence and weight distribution for the two different setups from Section 2.5. The plasticity parameters are the same as in Figure 2. The additive weight update rule is slower to converge since the weight updates are smaller for large input weights compared with the multiplicative rule. Figure 6D shows that in this specific setting, the synaptic weights do not need to be explicitly upper bounded. This holds because the input arrives in a random order and the causative effect of a single synapse is small. Figure 6D also shows that the additive (multiplicative) weight update rule converges to a bimodal (unimodal) distribution. Both uni- and bimodal distributions have been observed for earlier plasticity rules, whereas bimodal distributions are considered a sign of competition since some synapses being strong force others to be weak.

#### 3.2.4. Robustness of convergence

**Figure 7:**
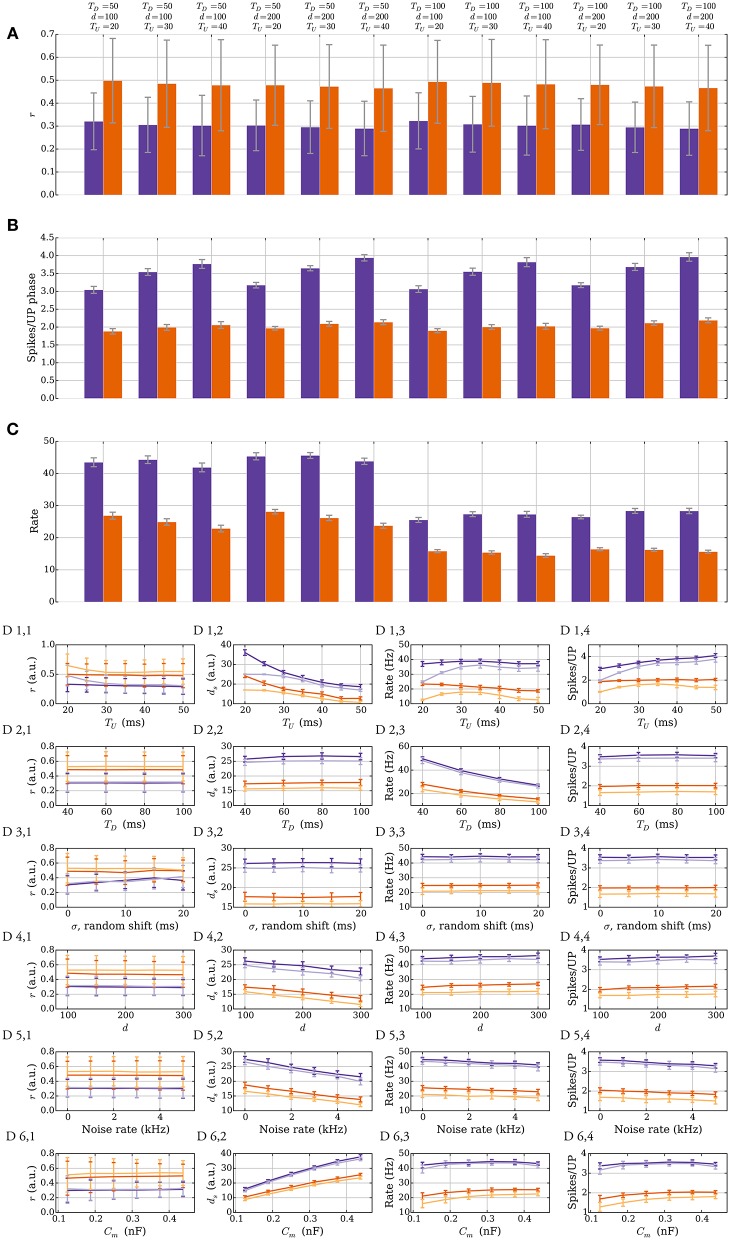
**Robustness against varying input parameters in the model with leak (Section 3.2.4). (A–C)** Results for two different sets of plasticity parameters corresponding to *r* = 1/3 (*purple*) and *r* = 1/2 (*orange*) in Table 3 are shown. Three parameters of the input are varied: the length of an UP phase *T*_*U*_; the number of input neurons *d*; and the length of a DOWN phase *T*_*D*_. **(A)** *r*, the expected time of the first postsynaptic spike within an UP phase scaled by the UP phase length. The plasticity parameters fix *r*; thus, varying the input parameters has a negligible effect on it. **(B)** Variations in the number of spikes per UP phase. The number of spikes per UP phase depends on *r*, but it also has a mild dependence on the refractory period and the reset potential, which explains the variations seen when varying *T*_*U*_. **(C)** Output rate variations. The rule does not fix the output rate of a neuron: the rate depends mainly on *T*_*U*_ and *T*_*D*_. **(D)** Single parameter variations. Variations for six different parameters are shown. The *darker colored curves* correspond to the same plasticity parameters as in the top three panels, and the *lighter colored curves* correspond to a setup with no positive feedback (*p*_*w*→*s*_ = 0). The *first row* is for *T*_*U*_, the *second* is for *T*_*D*_, the *third* is for random phase shifts of the input neurons, the *fourth* is for *d*, the *fifth* is for the noise rate ν, and the *sixth* is for the membrane capacitance (for details see Section 3.2.4). The *orange curves* start with *d*_*s*_ = 17 and the *purple curves* start with *d*_*s*_ = 25. Each data point in **(A–C)** is the mean of 40 trials and in **(D)** the mean of 10 trials. The error bars represent standard deviation estimates. For each trial, the process was simulated for 200 UP phases to reach the stable state and then the data points were collected over a continued simulation of 200 UP phases (*r*, rate, spikes per UP phase), or at the end (*d*_*s*_).

We show that the stability of the rule does not depend on the exact input parameters and that perturbations do not qualitatively affect it. Figure 7 illustrates this robustness in the model with leak. The different panels show two different plasticity parameter settings that correspond to *r* = 1/3 (*purple*) and *r* = 1/2 (*orange*) in Table 3. In Figures 7A–C, the lengths of UP and DOWN phases (*T*_*U*_ and *T*_*D*_), and the number of inputs *d* are varied. The process is first simulated for 200 UP phases starting with *d*_*s*_ = 25 such that the input weight reaches the stable state. The data in the figures is based on UP phases 201–400 in 40 trials. Figure 7A shows that the SLN rule fixes the relative spike time *r* of the postsynaptic neuron. The stable value of *r* mildly depends on *T*_*U*_, which arises from the choice of synaptic delay *t*_delay_ = 1 ms. To explain this, denote by *t*_1_ the spike time of the pre spike which triggers the postsynaptic spike at time *t*_2_. Any presynaptic neuron that spikes in the interval (*t*_1_, *t*_2_) receives a potentiation signal, which leads to a mild bias toward potentiation that is stronger for short UP phases. The variable *r* determines the number of spikes per UP phase (Figure 7B) since the number of spikes is inversely proportional to *r*. The number of spikes per UP phase has a mild dependence on *T*_*U*_ since the refractory period has a stronger effect for short UP phases and the reset potential makes repeated spikes easier. Figure 7C shows that the intrinsic homeostasis mechanism does not fix the rate of the output neuron since it is determined by *T*_*U*_ and *T*_*D*_.

Figure 7D shows the effects of single parameter variations. The *darker colored curves* correspond to the same plasticity parameters as in Figures 7A–C and the *lighter colored curves* correspond to a setting without positive feedback, i.e., when *p*_*w*→*s*_ = 0 (see Section 3.1.2 for a similar setting in the model without leak). Observe that most single parameter variations in this setting do not cause large plasticity undershooting. The main effects caused by *T*_*U*_ and *T*_*D*_ are covered above so we do not cover the first two rows here (the lower order effects of *T*_*U*_ on *r* are covered in the next paragraph). The third row corresponds to varying the phase shift of the inputs randomly (see Section 2.2). For that setting we replace *T*_*U*_ by *T*_*U*_ + σ in the definition of *r*. We see *r* increase due to a reduced input weight like we observed for more orderly input distributions in the model without leak (see Section 3.1.2). Rows four and five correspond to the number of input neurons and noise rate, respectively. Variations on both of these parameters have similar effects, as increasing these parameters helps activate the target neuron. Consequently, *d*_*s*_ must decrease to compensate (D 4,2 and D 5,2). The last row corresponds to varying the membrane capacitance *C*_*m*_. Increasing the capacitance decreases the leak conductance, but it also reduces the excitatory synaptic conductance, which makes it harder to activate the neuron. Despite large variations, *r* remains fixed.

To conclude this section, let us consider the lower order effects from *T*_*U*_, *d*, and noise strength on *r*. For simplicity assume that θ_*P*_ and θ_*D*_ are chosen such that a δ fraction of the strong (weak) synapses satisfies *m*(*t*) ∉ [θ_*D*_, θ_*P*_] in the stable state. For the corresponding input weight $d_{s}^{*}$, the parameters *p*_*s*→*w*_ and *p*_*w*→*s*_ satisfy

(27) $$\begin{array}{r} \left(d-d_{s}^{*}\right)\cdot \delta \cdot p_{w\to s}=d_{s}^{*}\cdot \delta \cdot p_{s\to w}. \end{array}$$

However, if we increase *T*_*U*_, the noise, or *d*, then $d_{s}^{*}$ will change to compensate such increase. If $d_{s}^{*}$ needs to decrease by *x* to obtain the correct value of *r*, then the left-hand side in Equation (27) increases by *xδp*_*w*→*s*_ whereas the right-hand side decreases by *xδp*_*s*→*w*_. Therefore, the number of strong synapses will not converge to exactly $d_{s}^{*}$ − *x*. However, if the interval [θ_*D*_, θ_*P*_] is large enough such that δ is small, then this effect is reduced.

#### 3.2.5. All-to-all vs. distinguished spikes

**Figure 8:**
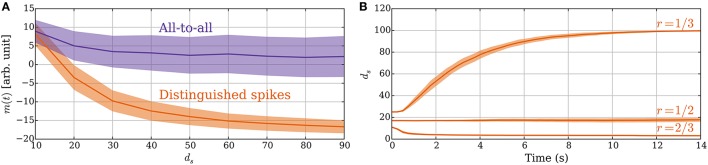
**(A)** Advantage of DSPs (Section 3.2.5). With DSPs in the model with leak, the variance of the memory trace is considerably smaller than for an all-to-all rule. The curves show the mean memory trace value when varying the number of strong inputs *d*_*s*_ in a static setting with *p*_*s*→*w*_ = *p*_*w*→*s*_ = 0. The *orange curve* corresponds to the SLN rule. The *purple curve* corresponds to a rule where all pre–post and post–pre spike pairs within an UP phase trigger learning signals. **(B)** Constant rate input at 40 Hz in the model with leak (Section 3.2.6). The two different sets of plasticity parameters from Table 3 are compared. For each setting, the number of strong synapses *d*_*s*_ is initially set in the stable state for the oscillating input. For the *r* = 1/3 parameters, all the synapses increase their weights since the synapses receive potentiation signals half of the time instead of one-third of the time, which they expect. For the *r* = 1/2 parameters, there is no such disagreement and for the *r* = 2/3 parameters it is the opposite and the input weight decreases to a point where activation becomes unreliable. In **(A)** the mean is taken after a 50-s-simulation over 10 trials, where in each trial samples are taken from all 100 synapses. In **(B)** the curves show means of 50 trials. In both panels the envelopes represent standard deviation estimates.

The principle of sampling the expected spike time of the postsynaptic neuron in an UP phase also applies if the synapse considers all pre/post and post/pre spike pairs within an UP phase to constitute potentiation and depression signals. In this section, we show the advantage of using a single DSP: it improves the signal-to-noise ratio. Furthermore, later postsynaptic spikes in an UP phase have more preceding presynaptic spikes in the UP phase and, thus, carry a weaker signal. Figure 8A compares the memory value for these two rules in a static setting where synapses have fixed weights. The figure shows the advantage of DSPs. They reduce the variance of the memory trace and increase its range, which makes the two different values of *d*_*s*_ more distinguishable. It may be noted that both versions of the rule perform similarly for small values of *d*_*s*_ because then only one postsynaptic spike is expected (i.e., both act as with DSPs). However, as *d*_*s*_ grows, and the postsynaptic rate increases the memory traces for the two rules start to differ. The difference arises from the fact that if the postsynaptic neuron spikes for example five times, then the update to the memory trace for DSPs is only −1 or 1 (for a single presynaptic spike), whereas it can range from −5 to 5 in the all-to-all setting. Furthermore, depression signals are more common in the DSP setting for large *d*_*s*_, whereas potentiation dominates in the all-to-all setting. This effect comes from the spike time distribution of the postsynaptic neuron within an UP phase.

#### 3.2.6. Fixed rate input

So far we assumed that the input signal oscillates between high and low states of activity. One may also ask, however, what happens if the input rate is constant, which is a common assumption in modeling studies (Burkitt, 2006a). In this regard, the behavior of the SLN rule is determined by the ratio of potentiation and depression signals as before. However, for constant input rate the rule does not have a frame of reference to measure the input strength anymore. The ratio of potentiation and depression signals is, therefore, determined by the parameters *T*_early_ and *T*_late_. If we choose the parameters θ_*P*_, θ_*D*_ such that the memory trace lies entirely within the interval [θ_*D*_, θ_*P*_] for constant input rate, then the weights do not change. However, if a larger fraction of the memory trace distribution lies below θ_*D*_ than above θ_*P*_, then the total input weight decreases up to a point where activation of the neuron becomes unreliable. For the opposite case, all the synapses get strengthened, which is not desirable. Figure 8B illustrates this by showing how the system responds to constant input for three different sets of plasticity parameters. For parameters that result in *r* > 1/2, the weights decrease to a point where the target neuron shows almost no reaction to the input. Indeed, this seems a reasonable response as such an input does not carry any information. On the other hand, for parameters that result in *r* < 1/2 the weights and the firing rate of the target neuron grow to the maximum value, which is undesirable if the activity of the neuron should be limited. These observations indicate that the parameters should fix *r* to be at most 1/2, which corresponds to several spikes of a neuron in an UP phase (≈ 1/*r*) and is in good agreement with experimental findings (Connors and Gutnick, 1990). Furthermore, the synapses should not expect disproportionally more potentiation signals than depression signals.

## 4. Discussion

There are two forms of homeostasis prominently discussed in the literature. First, there is the concept of normalization (or scaling) of total input weights with respect to a target total weight (Von der Malsburg, 1973). Second is the concept of rate normalization, where rate of the postsynaptic neuron should stay within a target regime. The latter option is regarded to be more plausible than the former, insofar as it can be implemented locally at the level of a single synapse so that the computation is restricted to the sole information available at that synapse. In contrast, the first option requires computation of the total input weight which was not considered a synapse specific computation but rather a neuron specific computation (Zenke et al., 2013). In this paper, we introduce a third option: a fast-acting local homeostasis mechanism that normalizes the expected spike time of a neuron in an UP phase of an oscillatory input rate function. Two independent studies have identified the order of the time scale for fast homeostasis to be seconds (El Boustani et al., 2012; Zenke et al., 2013), which is well within the parameter regimes of the SLN rule presented in this paper (for a recent review see Zenke and Gerstner, 2017). Furthermore, normalization is applied on a fast time scale in most, if not all, computational studies (Chistiakova et al., 2015). The fact that the SLN rule is Hebbian challenges the belief that regulation of neuronal excitability is difficult if synapses are modified independently by such rules (Abbott and Nelson, 2000). Furthermore, for long UP phases the rule results in input weight normalization which challenges the belief that estimating the input weight cannot be done through local synaptic computation. Moreover, the rule is energy-efficient in the sense that the synaptic weights do not change in the stable state for parameters favoring stability. In particular, if the postsynaptic neuron is spiking with the correct expected relative spike time, then the homeostasis mechanism does not intervene. Hence, the weights do not converge to a unique distribution.

### 4.1. Related literature

#### 4.1.1. Related metaplasticity rules

The SLN rule bears some resemblance to the metaplasticity rule proposed by Brader et al. (2007). Common to both rules is a synapse specific memory trace represented by a real value which is updated for certain spike events. Other metaplasticity rules depend on discrete states within the synapses such as the synaptic integration rule by Elliott and Lagogiannis (2012) which can be considered a multimodal version of the cascade rule by Fusi et al. (2005). However, these metaplasticity rules have not been studied in the context of SLN for UP/DOWN state input activity where, in particular, the fast and robust weight convergence for such activity has not been demonstrated before.

#### 4.1.2. Volley-like and repeating input patterns

The effect of standard STDP on spike timing was studied and analyzed in detail in the context of volley-like input spike distributions in Gerstner and Kistler (2002, Chapter 12.2). Furthermore, if the relative effect of potentiation is reduced then STDP leads to convergence of the input weights to a stable distribution for random input activity (Van Rossum et al., 2000). For both these cases, the synaptic weights perform random walks in the stable setting whereas the SLN rule provides a stronger stability guarantee since it implicitly fixes the weights at equilibrium and the weight distribution at convergence is not unique. Hence, the SLN rule is economical in the sense that synaptic weights are only changed when it is necessary which is in agreement with other studies since not all spike pairs necessarily lead to a weight change (Yger and Gilson, 2015).

Other theoretical studies have considered repeating spike inputs as the ones presented in Section 3.1.2. Guyonneau et al. (2005) studied standard STDP for repeated stimulation of a neuron with the exact same spike pattern. Through such repeated exposure the postsynaptic neuron becomes a coincidence detector for the given pattern. Further studies showed that this feature is robust to noise (Masquelier et al., 2008, 2009).

#### 4.1.3. Other activity dependent homeostasis mechanisms

Perhaps the most prominent example of homeostasis in plasticity models is the BCM rule (Bienenstock et al., 1982). The weight update in the BCM rule normalizes postsynaptic rate by using a threshold which varies slowly with the postsynaptic rate. Nearest-neighbor STDP has been shown to perform a simplified version of this operation, i.e., without the sliding threshold, when pre- and postsynaptic activity is weakly correlated (Izhikevich and Desai, 2003).

Other studies have focused more directly on preventing single weights from being driven to extreme values. Gütig et al. (2003) presented a plasticity model where the plasticity rule is weight dependent and, therefore, leads to an implicit upper bound on synaptic weights through a mixture of additive and multiplicative weight changes. Babadi and Abbott (2010) had an even more implicit approach by shifting the effective STDP temporal window by roughly 2 ms such that pre/post pairs that are close in time lead to depression instead of potentiation. This effect works against synapses with a large causative spiking effect. It may be noted that the same principle can also be applied for the SLN rule.

Another variable which could be controlled through homeostasis is the type of activity in a neural network. In this regard, STDP is known to sustain a stable background state in balanced networks (Kempter et al., 1999, 2001; Song et al., 2000; Van Rossum et al., 2000; Rubin et al., 2001) and to regulate activity (Kempter et al., 1999, 2001; Song and Abbott, 2001; Pfister et al., 2006; Watt and Desai, 2010) but these models have been criticized for parameter fine-tuning (for a review see Chistiakova et al., 2015). Theoretical investigations showed that fast detection of postsynaptic rate changes can solve the problem (Zenke et al., 2013; Yger and Gilson, 2015) but these studies do not consider the scenario of UP and DOWN phases.

It is plausible that homeostatic plasticity is controlled by more than a single mechanism (Tononi et al., 1999; Watt and Desai, 2010) as many alternatives, like we discussed, which have a homeostatic effect on various parameters of neurons or network activity have been proposed (Bienenstock et al., 1982; Rabinowitch and Segev, 2006; Vogels et al., 2011; Remme and Wadman, 2012). It is known that homeostatic plasticity is affected by a complex web of signaling processes many of which are likely undiscovered (Pozo and Goda, 2010) and which might even be shared between, or belong to, different homeostasis mechanisms. As an example, weight normalization is considered biologically plausible because a neuron might have finite resources dedicated to maintaining its synapses, but there is a lack of experimental evidence to support this.

### 4.2. Biophysical bases for memory traces

For the SLN rule, we require the synapses to hold a trace of learning signals from the past. Some previous learning models depend on a synapse-specific memory trace that represents global information shared by all or many input synapses of a neuron (Brader et al., 2007; Urbanczik and Senn, 2009; Brea et al., 2013, 2016; Urbanczik and Senn, 2014). Several hypotheses propose that some form of memory can reside in a synapse. Min and Nevian (2012) suggest that astrocytes could act as a memory buffer to store previous coincident spike events. Astrocytes could further provide the signal that determines if a synapse should increase or decrease its weight (De Pittà et al., 2016; De Pittà and Brunel, 2016). Another candidate that has been proposed to represent a long-lasting memory trace of past synaptic activity is calcium/calmodulin-dependent protein kinase II (CaMKII) (Hell, 2014). This protein kinase can assume two stable states depending on whether it is phosphorylated (active) or not (auto-inhibited). Earlier results have shown that LTP induction induces a persistent translocation of CaMKII to synaptic spines, causing it to be considered as a form of a long-term memory trace (Otmakhov et al., 2004). Other more short-lived memory traces have also been proposed such as transient neurotransmitter concentrations, like dopamine, which can extend the window of potentiation in STDP and is supported by experimental evidence (Zhang et al., 2009), and the local membrane potential at a synapse (Urbanczik and Senn, 2014).

### 4.3. Rhythmic input

The intrinsic homeostasis effect of the SLN rule is designed for input activity that switches between high and low states. Oscillations of activity are ubiquitous in the brain (Reyes, 2003; Buzsáki and Draguhn, 2004), but their exact functional role is still not fully understood. The most prominent hypothesis of the functional role of oscillations in brain activity is the “communication-through-coherence” hypothesis, see the reviews in Fell and Axmacher (2011) and Thut et al. (2012). This hypothesis posits that neural populations communicate through phase-locked oscillations. If the subthreshold potentials of the two populations are phase locked, then the source population can activate the target if it is in an UP state when the signal arrives, which is considered to lead to long-term potentiation. Correspondingly, if the target population is in a DOWN state, then the activation becomes harder and is believed to lead to long-term depression. Recent evidence indicates that retrieval of information in the hippocampus is discretized with respect to slow-wave gamma oscillations and sharp-wave ripple events, which are recognized as the most synchronous patterns in the brain (Pfeiffer and Foster, 2015). However, synchronous and rhythmic activity is also observed during sleep and then the duration of UP and DOWN phases is typically in the order of seconds (Steriade et al., 2001; Jercog et al., 2016). Even though these observations were first made some decades ago (Steriade et al., 1993a,b,c) the functional role of UP and DOWN phases is still not apparent (Vyazovskiy and Faraguna, 2014).

### 4.4. Conclusion

We have shown that Hebbian learning can be intrinsically stable through postsynaptic spike latency normalization in the context of activity that alternates between UP and DOWN phases. Remarkably, the mechanism is fast since only a few cycles of UP and DOWN phases are necessary to normalize the postsynaptic spike latency. This highlights a potential functional role of UP and DOWN phases, that is, they provide a frame of reference in which synapses can measure postsynaptic spike latency which results in a homeostatic effect on postsynaptic activity. In particular, for long UP phases, spike latency normalization is equivalent to input weight normalization which was considered not to be possible locally at the synapse level. Furthermore, the total input weight to which the SLN rule converges is inversely related to the length of the UP phase for short UP phases and is independent of the length of the UP phase for long UP phases. This feature is in agreement with the synaptic homeostasis hypothesis (Tononi and Cirelli, 2003, 2006, 2014) which proposes that synaptic weights are downscaled during slow-wave sleep when long UP and DOWN phases are typically observed (Steriade et al., 2001). Future work will involve the study of spike latency normalization in recurrent and feed-forward networks. Denève and Machens (2016) have shown that UP and DOWN phases can be a characteristic of simulated recurrent networks. Our preliminary results indicate that in feed-forward networks the rule increases synchrony with network depth and the expected number of spikes in an UP phase converges to approximately 1/*r* for deep layers and short UP phases.

## Author contributions

The project idea came from AS and the analysis was performed in collaboration by all the authors. HE did all simulations, prepared the figures and wrote a first draft of the manuscript. All authors helped revise the manuscript and everyone approved the final version of it.

## Funding

HE was supported by grant no. 200021 143337 of the Swiss National Science Foundation. MG was supported by CNPq grant no. 248952/2013-7.

### Conflict of interest statement

The authors declare that the research was conducted in the absence of any commercial or financial relationships that could be construed as a potential conflict of interest.
