# Supplementary material for: A Model of Fast Hebbian Spike Latency Normalization
Source: Front Comput Neurosci. 2017 May 15;11:33. doi: 10.3389/fncom.2017.00033 (PMC5430963; doi:10.3389/fncom.2017.00033)
Supplement: Supplementary file 1 [file Presentation1.pdf]

A model of fast Hebbian spike latency normalization – Supplementary material

Hafsteinn Einarsson[1],Marcelo Matheus Gauy[1],
Johannes Lengler[1] and Angelika Steger[1,2]

[1]Department of Computer Science
ETHZ, 8092 Zürich, Switzerland

[2]Collegium Helveticum
Zürich, Switzerland

A Derivation of Analytical Results

A.1 Theoretical tools

To analyze the model without leak, we use Bernoulli and binomially distributed random variables. A Bernoulli random variable X takes the value 1 with probability p and 0 with probability $1-p$ for $0 \leq p \leq 1$. Denote this by $X \sim Be(p)$. A binomial random variable Y, with parameters n and p, is a sum of n independent Bernoulli variables with success probability p. Denote this by $Y \sim Bi(n,p)$. Bernoulli random variables are used to model the type of learning signal a synapse receives. The binomial distribution is used in two contexts, to sum up the Bernoulli variables in the memory from the model without leak and to sum up the total input weight in a volley when synapses are probabilistic.

In the analysis, the following two results are used:

Theorem 1 (Chernoff-bounds, formulation from [2, Theorems 4.4 and 4.5])**.** *Let* $X_1 \sim Be(p_1), \ldots, X_n \sim Be(p_n)$ *be independent Bernoulli distributed random variables, and let* $X = \sum_{i=1}^{n} X_i$*. Then, the following inequalities hold for* $\varepsilon \geq 0$*, and* $\mu \equiv \mathbb{E}[X] = \sum_{i=1}^{n} p_i$*.*

$$\Pr[X \geq (1+\varepsilon)\mu] \leq e^{-\mu\varepsilon^2/3}, \text{ and}$$
$$\Pr[X \leq (1-\varepsilon)\mu] \leq e^{-\mu\varepsilon^2/2}.$$

Theorem 2 (Variable Drift [1, Theorem 4.6])**.** *Let* $(X_t)_{t\geq 0}$ *be a sequence of random variables over a state space* $0 \in S \subseteq \mathbb{R}_0^+$ *such that* $s_{\min} \equiv \inf\{x \in S \mid x > 0\} > 0$*. Assume further that* $X_0 = s_0$ *for some* $s_0 \in S$*. Let* T *be the random variable that denotes the earliest point in time* $t \geq 0$ *such that* $X_t = 0$*. Suppose there is an increasing function* $h : \mathbb{R}^+ \to \mathbb{R}^+$ *such that for all* $x \in S \setminus \{0\}$*,*

$$\mathbb{E}[X_t - X_{t+1} \mid X_t = x] \geq h(x),$$

then, for all x*,*

$$\mathbb{E}[T] \leq \frac{s_{\min}}{h(s_{\min})} + \int_{s_{\min}}^{s_0} \frac{1}{h(u)} du.$$

A.2 Scaling of the memory trace with synaptic parameters

In this section, the exact derivation of how to choose the memory size as in Equation (19) such that at most a δ fraction of the synapses change their weight in the stable state is given. Recall that in the model without leak for $\varepsilon > 0$, and some value of d_s, for which one wants an upper bound on stability, the threshold parameters are set as in Equation (18). The requirement from above on limiting weight changes in the stable state translates to the following

two inequalities:

$$\text{if } w(t) = 1 \quad \Pr\left[m(t) \le \theta_D\right] = \Pr\left[\text{Bin}(M, p_{\text{early}}(d_s)) \le \theta_D M\right] \le \delta \tag{1}$$
$$\text{if } w(t) = 0 \quad \Pr\left[m(t) \ge \theta_P\right] = \Pr\left[\text{Bin}(M, p'_{\text{early}}(d_s)) \ge \theta_P M\right] \le \delta. \tag{2}$$

By Theorem 1

$$\Pr\left[\text{Bin}(M, p_{\text{early}}(d_s)) \le \theta_D M\right] \le e^{-\varepsilon^2 M p_{\text{early}}(d_s)/3} \text{ and} \tag{3}$$
$$\Pr\left[\text{Bin}(M, p'_{\text{early}}(d_s)) \ge \theta_P M\right] \le e^{-\varepsilon^2 M p'_{\text{early}}(d_s)/3}. \tag{4}$$

Since $p'_{\text{early}}(d_s) \le p_{\text{early}}(d_s)$ it suffices to choose M such that $e^{-\varepsilon^2 M p'_{\text{early}}(d_s)/3} = \delta$. Solving this equation for M yields

$$M = \frac{3\log(\delta^{-1})}{\varepsilon^2 p'_{\text{early}}(d_s)}. \tag{5}$$

To see how this upper bound scales with the size of the stable region for an absolute deviation from d_s, we set $\varepsilon = x/d_s$ where x is a positive integer. Plugging $\varepsilon = x/d_s$ into Equation (32) yields

$$M = O\left(\frac{(d_s)^3 \log(\delta^{-1})}{x^2 \theta_v}\right). \tag{6}$$

A.3 Convergence

We derive an upper bound for the expected number of weight updates until the input weight is close to the stable state. Assume that initially $d_s > (1+2\varepsilon)d_s^*$ and denote by T the number of weight updates until $d_s < (1+2\varepsilon)d_s^*$. We show that the expected value of T is upper bounded by $\frac{C}{p_{s\to w}} \cdot \log(d_s - (1+2\varepsilon)d_s^*)$, where C is a constant which depends on ε.

Let $\varepsilon < 1/4$ be the same as in Equation (18) for θ_P and θ_D. Assume that

$(d - d_s^*)p_{w \to s} = \Theta(d_s^* p_{s \to w})$ such that potentiation is balanced with excitation in the stable state. For simplicity, assume that the synapses are deterministic ($p_r = 1$). Furthermore, assume that the synapse is strong, i.e., $w_{vu} = 1$. For convenience, define $\mu_1 \equiv \frac{\theta_v}{d_s} M$. The probability of the event $m(t) \leq \theta_D$ can be lower bounded as follows:

$$
\begin{aligned}
\Pr\left[m(t) \leq \theta_D\right] &\geq \Pr\left[\mathrm{Bin}(M, p_{\mathrm{early}}(d_s)) \leq (1-\varepsilon)\frac{\theta_v}{d_s^*} M\right] \\
&= \Pr\left[\mathrm{Bin}(M, \theta_v/d_s) \leq \ (1-\varepsilon)\mu_1 \frac{d_s}{d_s^*}\right] \\
&\overset{(1)}{\geq} \Pr\left[\mathrm{Bin}(M, \theta_v/d_s) \leq \ (1+\varepsilon/2)\mu_1\right] \\
&\overset{(2)}{\geq} 1 - e^{-\varepsilon^2 \mu_1 / 12} \\
&\equiv f(\varepsilon, M),
\end{aligned}
$$

where in (1) the inequalities $\varepsilon < 1/4$ and $\frac{d_s}{d_s^*} \geq (1+2\varepsilon)$ were used and in (2) Theorem 1 was used. Define $\mu_0 \equiv \frac{\theta_v}{d_s^*+1} M$. For $w_{vu} = 0$; similarly, as above

$$
\begin{aligned}
\Pr\left[m(t) \geq \theta_P\right] &= \Pr\left[m(t) \geq (1+\varepsilon)\frac{\theta_v}{d_s^*+1} M\right] \\
&= \Pr\left[m(t) \geq (1+\varepsilon)\mu_0 \frac{d_s+1}{d_s^*+1}\right] \\
&\overset{(1)}{\leq} \Pr\left[m_{uv} \geq (1+2\varepsilon)\mu_0\right] \\
&\overset{(2)}{\leq} e^{-4\varepsilon^2 \mu_0 / 3} \\
&\equiv g(\varepsilon, M),
\end{aligned}
$$

where in (1) the inequality $\frac{d_s+1}{d_s^*+1} \geq 1 + \varepsilon$ was used and in (2) Theorem 1 was used. Assuming $d_s \geq (1+2\varepsilon)d_s^*$, the expected absolute weight drift, the

difference of Equations (15) and (16), can be lower bounded as follows:

$$\mathbb{E}\left[|\Delta_{s\to w}(d_s) - \Delta_{w\to s}(d_s)|\right] \geq f(\varepsilon, M)\cdot d_s \cdot p_{s\to w} - g(\varepsilon, M)\cdot (d - d_s)\cdot p_{w\to s}. \quad (7)$$

If $d_s \geq d_s^*$, then $p_{w\to s}$ was chosen such that $(d - d_s)p_{w\to s} = O(d_s p_{s\to w})$. By increasing M, $f(\varepsilon, M)$ can be made arbitrarily close to 1 and $g(\varepsilon, M)$ arbitrarily close to 0. Therefore, by choosing $M = M(\varepsilon, (d - d_s^*)p_{w\to s})$ large enough, such that

$$f(\varepsilon, M) > 1 - \varepsilon \quad \text{and} \quad g(\varepsilon, M)\cdot (d - d_s^*)p_{w\to s} < \varepsilon d_s^* p_{w\to s} \quad (8)$$

one can lower bound the amount of progress in a weight update toward the stable state by

$$\mathbb{E}\left[|\Delta_{s\to w}(d_s) - \Delta_{w\to s}(d_s)|\right] \geq (1 - 2\varepsilon)\cdot p_{s\to w} d_s. \quad (9)$$

Denote by $d_s(t)$ the number of strong synapses after t learning rounds and set $d_s'(t) = \max\{1, d_s(0) - (1 + 2\varepsilon)d_s^*\}$. Set $T = \min_t\{t \mid d_s'(t) = 0\}$ which is the time needed to hit the interval around d_s^* in the shifted process, d_s'. By Theorem 2, the variable drift theorem,

$$\begin{aligned}\mathbb{E}[T] &\leq \frac{1}{(1-2\varepsilon)\cdot p_{s\to w} d_s^*} + \int_1^{d_s'(t)} \frac{1}{(1-2\varepsilon)\cdot p_{s\to w} s} ds \\ &= \frac{1}{(1-2\varepsilon)\cdot p_{s\to w}} \cdot \left(\frac{1}{d_s^*} + \log(d_s'(t))\right)\end{aligned}$$

The case for $d_s = (1 - 2\varepsilon)d_s^*$ follows an analogous argument.

A.4 Validity of the homeostatic mechanism regardless of the random order assumption

In this section, we prove that the intrinsic homeostasis mechanism works for any probability distribution on the set of all orderings. More precisely, if in each volley an ordering is drawn from this distribution, then the input weight remains bounded. This result shows that the mechanism provides negative feedback for arbitrary input distributions. This result essentially follows from the fact that only a few synapses can remain strong because only θ_v of them are needed to activate the target neuron.

For large input weight, the expected weight drift is negative. Denote a synapse between neuron u and v by (u, v), and denote their weight by w_{uv}. In what follows, d_s^* denotes as usual the stable state for the standard process when the input order is chosen uniformly at random.

For a set S, denote by $|S|$ the number of elements it contains. To study this setting, let $E_1 \equiv \{(u, v)|w_{uv} = 1\}$ be the set of strong synapses and for $e_{uv} \in E_1$, let X_{uv} be an indicator random variable for the event that u spikes before v in this volley (X_{uv} is 1 if the event occurs and 0 otherwise). Furthermore, let $p_{uv} = \Pr[X_{uv} = 1]$ be the success probability. Then, by linearity of expectation and the fact that only θ_v of the strong synapses are early,

$$\sum_{(u,v)\in E_1} p_{uv} = \mathbb{E}\left[\sum_{(u,v)\in E_1} 1 - X_{uv}\right] = \theta_v. \qquad (10)$$

For $0 < \varepsilon < 1/2$, assume $d_s > \frac{d_s^*}{1-2\varepsilon}$, i.e., the input weight, is too large. Strong synapses with $p_{uv} > (1 - 2\varepsilon)\frac{\theta_v}{d_s^*} = p^*$ have a chance to retain their weight whereas other strong ones will have $m(t) < \theta_D$ with high probability by Theorem 1. To formalize this, denote by $E_{\text{fast}} \subseteq E_1$ the synapses that have $p_{uv} > p^*$ and by $E_{\text{slow}} \subseteq E_1$ those who have $p_{uv} \leq p^*$. Furthermore, let $p_{\text{fast}} \equiv |E_{\text{fast}}|^{-1} \sum_{(u,v)\in E_{\text{fast}}} p_{uv}$ and $p_{\text{slow}} \equiv |E_{\text{slow}}|^{-1} \sum_{(u,v)\in E_{\text{slow}}} p_{uv}$ so that by

Equation (37) it follows that

$$|E_{\text{fast}}|p_{\text{fast}} + (d_s - |E_{\text{fast}}|)p_{\text{slow}} = \theta_v. \tag{11}$$

Using $p^* < p_{\text{fast}}$, one obtains the following upper bound on the number of fast synapses:

$$|E_{\text{fast}}| < \frac{\theta_v - d_s p_{\text{slow}}}{p^* - p_{\text{slow}}}. \tag{12}$$

Take the derivative w.r.t. p_{slow} to maximize the upper bound on $|E_{\text{fast}}|$; that is,

$$\frac{d}{dp_{\text{slow}}} \frac{\theta_v - d_s p_{\text{slow}}}{p^* - p_{\text{slow}}} = \frac{\theta_v - d_s p^*}{(p^* - p_{\text{slow}})^2}. \tag{13}$$

For $d_s > \frac{\theta_v}{p^*} = \frac{d_s^*}{1-2\varepsilon}$, the upper bound for $|E_{\text{fast}}|$ is maximized for $p_{\text{slow}} = 0$. Therefore,

$$|E_{\text{fast}}| < \frac{d_s^*}{1 - 2\varepsilon}. \tag{14}$$

Since $d_s > \frac{d_s^*}{1-2\varepsilon}$, there is high probability of at least $d_s - \frac{d_s^*}{1-2\varepsilon}$ slow synapses that will turn weak with probability $p_{s\to w}$ in the next weight update. Once at most d_s^* synapses (and at least θ_v) among the fastest ones are strong, the weight cannot increase further and it stays upper bounded with high probability.

References

[1] Daniel Johannsen. *Random Combinatorial Structures and Randomized Search Heuristics.* PhD thesis, Universität des Saarlandes, 2010. Available online at http://scidok.sulb.uni-saarland.de/volltexte/2011/3529/pdf/Dissertation_3166_Joha_Dani_2010.pdf.

[2] Michael Mitzenmacher and Eli Upfal. *Probability and computing: Randomized algorithms and probabilistic analysis*. Cambridge University Press, 2005.
